# Effectiveness of interventions for informal caregivers of people with end-stage chronic illness: a systematic review

**DOI:** 10.1186/s13643-024-02641-x

**Published:** 2024-09-28

**Authors:** K. McGuigan, G. Laurente, A. Christie, C. Carswell, C. Moran, M. M. Yaqoob, S. Bolton, R. Mullan, S. Rej, P. Gilbert, C. McKeaveney, C. McVeigh, C. Tierney, J. Reid, I. Walsh, T. Forbes, H. Noble

**Affiliations:** 1 School of Nursing and Midwifery, Queen’s University Belfast, 97 Lisburn Road, Belfast, BT9 7BL UK; 2 Western Health and Social Care Trust, Londonderry, UK; 3 Barts Health NHS Trust, London, UK; 4 Northern Health and Social Care Trust, Antrim, UK; 5 Faculty of Medicine and Health Sciences, McGill University, Montréal, Canada; 6 Northern Ireland Kidney Patient Association, Belfast, UK; 7 School of Medicine, Dentistry and Biomedical Sciences, Queen’s University Belfast, Belfast, UK

**Keywords:** Caregivers, Advanced, Chronic illness, Intervention, Psychosocial, Systematic review

## Abstract

**Background:**

People living with advanced, non-malignant chronic conditions often have extensive and complex care needs. Informal or family caregivers often provide the care and support needed by those with advanced chronic conditions at home. These informal caregivers experience many challenges associated with their caring role, which can impact their own wellbeing. Whilst there is growing evidence around the impact on carers, guidance on support for informal caregivers of patients with advanced, non-malignant, chronic conditions is lacking, with little evidence available on effective psychosocial carer interventions. This systematic review explored existing interventions for caregivers of those with advanced, non-malignant, chronic illness, in order to assess the effectiveness of these interventions in improving psychosocial outcomes.

**Methods:**

Electronic databases, Medline, CINAHL, EMBASE, and PsycINFO, were searched up to the end of March 2023. Studies meeting the inclusion criteria, focusing on interventions to improve psychosocial outcomes, such as depression, anxiety, quality of life, and caregiver burden, in this cohort of caregivers were included. Data were extracted regarding study setting, design, methods, intervention components, and outcomes. Risk of bias and quality assessment were conducted.

**Results:**

A total of 5281 articles were screened, ultimately identifying 12 studies for inclusion, reported in 13 publications. A narrative synthesis revealed mixed results. Psychosocial interventions resulted in more significant improvements in psychosocial outcomes than psychoeducational or support interventions, with interventions for carer-patient dyads also reflecting more positive outcomes for caregivers. Evidence-based interventions, guided by an appropriate theoretical model, were reportedly more effective in improving caregiver outcomes. Differences in outcomes were related to intervention development, design, delivery, and outcome assessment.

**Conclusions:**

This review, to our knowledge, is the first to explore the effectiveness of interventions in improving psychosocial outcomes for caregivers of those with advanced, non-malignant, chronic conditions. The review highlights the need for more robust, sufficiently powered, high-quality trials of evidence-based interventions for caregivers of people with advanced chronic illness. Optimal intervention duration and frequency of sessions are unclear and need further exploration.

**Supplementary Information:**

The online version contains supplementary material available at 10.1186/s13643-024-02641-x.

## Introduction

The number of older adults with advanced, non-malignant conditions in need of end-of-life care exceeds those with malignant conditions [1]. This is expected to grow due to an aging population and the rise in the number of people with chronic illness [2]. As such, it is anticipated that there will be increased demand from people living with chronic conditions who are frail and require complex care [3]. Current UK figures estimate around 1 in 8 people living here are carers (6.5 ~ 6.8 million) [4, 5]. Informal caregivers provide the patient with care and support in all aspects of advanced illness. This may include provision of emotional support, support with daily activities, providing physical care, completing household tasks, cooking, collecting prescriptions, monitoring medications and medication adherence, monitoring symptoms, possibly even actively participating in care planning and decision-making, and often acting as an advocate for the patient [6, 7].

As such, informal carers in the UK serve not only to provide care, but also to reduce the burden on the health service; however, these caregivers are often unsupported placing them at risk of burnout [8]. Caregiver need remains largely unaddressed with research to date in this area generally guided by work in cancer care [1, 9]. Caregiving in advanced chronic disease is demanding in nature [3, 10], impacting the caregiver’s physical, psychological, emotional, and social wellbeing [11, 12]. Advanced ‘chronic and uncertain conditions’ present a huge challenge for informal carers [13] p. 2). Caregivers may have acted in their capacity as caregiver for a long period of time [14] with patient needs changing over time, and advancing illness bringing different or increasing symptoms [13]. Caregiver burden in chronic illness will generally increase as the condition progresses [12]. Informal caregivers cannot manage this burden alone; they need support, guidance, knowledge, and skills to manage the complex care needs of advanced chronic illness [14, 15].

Caregiving can take its toll on the caregiver, affecting their physical, psychological, and emotional wellbeing [10–12]. Providing supportive interventions to address caregiver needs and challenges in advanced, non-malignant, chronic illness is essential, as left unaddressed, these challenges can affect condition management for patients, serving to increase the complexity of chronic condition management [10]. Non-malignant refers to a condition which is not cancerous, such as neurological conditions, coronary heart disease, or kidney disease [16]. It is argued that intervention among this cohort of caregivers is essential to guard against caregiver burden and burnout, and is becoming increasingly more urgent in light of an aging population [17]. There is a growing body of literature highlighting the experiences, burden, and negative impact of caring on caregivers for those with advanced chronic illness, (e.g. [10, 17, 18]). However, supportive interventions for these caregivers remain underdeveloped [10, 12]. The literature highlights the need for improved provision focused on ‘developing tools to help caregivers cope and manage their own needs’ [17]p. 9).

Research to date has highlighted the needs of caregivers in advanced illness, with clear demand for support responsive to their emotional and psychosocial needs, information on condition management and practical aspects, as well as advice on self-care [18, 19]. There is a call from caregivers that this support should be more proactive in nature, responding at an earlier stage rather than when the situation becomes unmanageable [19, 20]. There is agreement within the literature that interventions should ensure ‘prioritisation of psychological impact from caring’ [4] p. 356), particularly considering the increased incidence of depression, anxiety, stress, and burden among caregivers for those with advanced chronic illness [1, 4, 19]. The need to explore effective interventions for this group of caregivers is increasingly acute due to the growth of chronic illness and our aging population, both of which will be indicative of future demand for care [21]. Given the lack of existing collated information on effective intervention for carers, the aim of this systematic review was to explore the effectiveness of interventions in improving psychosocial outcomes for caregivers of those with advanced, non-malignant, chronic conditions.

## Materials and methods

### Registration

This systematic review followed the Preferred Reporting Items for Systematic Reviews and Meta-Analyses (PRISMA) guidelines [22]. The review was registered, and accepted, in the international Prospective Register of Systematic Reviews (PROSPERO) [CRD42021279151].

### Search strategy

Four electronic databases, Medline, CINAHL, EMBASE, and PsycINFO, were searched up to 31st March 2023, with no date restrictions applied. Appropriate key words and medical subject heading terms (MeSH) for studies, that were relevant to psychosocial outcomes in caregivers for patients with end-stage/advanced chronic illness, were developed and verified by the research team (see Supplementary Materials Table 1).

### Eligibility

The review utilised the Population, Intervention, Comparison, Outcomes and Study design (PICOS) search tool for inclusion and exclusion criteria.

Inclusion criteria:

P — Adult, informal caregivers for individuals with end-stage/palliative/advanced chronic conditions (≥ 50% of sample to fit this patient cohort in mixed samples).

I — Targeting caregiver psychosocial outcomes to include quality of life (QoL), depression, anxiety, caregiver burden; or secondary outcomes of interest including distress, stress, and self-efficacy.

C — Comparator group not necessary. Baseline and follow-up data (pre-post intervention at a minimum) to evidence any changes affected by intervention in caregivers.

O — Quantitative (can be as part of mixed methods design); QoL, depression or/and anxiety, caregiver burden; or secondary outcomes of interest as above. Reported as standardised mean difference (SMD) from baseline.

S — Randomised controlled trials (RCTs), or intervention/non-traditional/quasi-experimental trials, e.g. pre and post intervention testing, non-randomised with control.

Exclusion criteria are as follows: dissertations; study protocols; case studies; studies with incomplete data; cross-sectional studies were excluded. Studies were also excluded if they focused on the following: patient rather than caregiver outcomes; caregiver involvement in patient management only; caregivers for children; malignant conditions; or qualitative outcomes.

### Study selection and data extraction

All records identified through database searches were imported into Covidence, online systematic review management software [23]. Duplicates (*n* = 1134) were removed. All remaining 5281 titles and abstracts were screened by the lead author (KMG) and independently by two researchers (GL and AC). Full-text (*n* = 67) review was completed by the lead author (KMG) with two independent researchers (GL and AC) sharing the full-text screening to ensure agreement. Any disagreements were to be resolved by a fourth researcher (CC); however, no disagreements arose.

Data were extracted, independently, by three reviewers (KMG, GL, AC). For the included studies, key data were extracted, including the following: study details/characteristics (first author, country and year of publication), study design, population, intervention description/components, relevant outcome measures, and intervention effects.

### Risk of bias and quality assessment

Risk of bias was assessed using the Cochrane Risk of Bias 2.0 (RoB 2.0) [24]. RoB 2.0 allows for the assessment of risk of bias across six domains, with resulting low, unclear, or high risk of bias. The quality of RCTs in this review was assessed using this tool. For the remaining trials, the Joanna Briggs Institute reviewer manual was used to assess the risk of bias and quality across nine domains [25].

### Data synthesis

Due to the wide variation in clinical population, outcome measures, and intervention design, a narrative synthesis of the data was conducted, in line with the Centre for Reviews and Dissemination guidance [26]. Study characteristics, intervention characteristics, quality, and findings are reported.

## Results

### Selection of studies

**Fig. 1:**
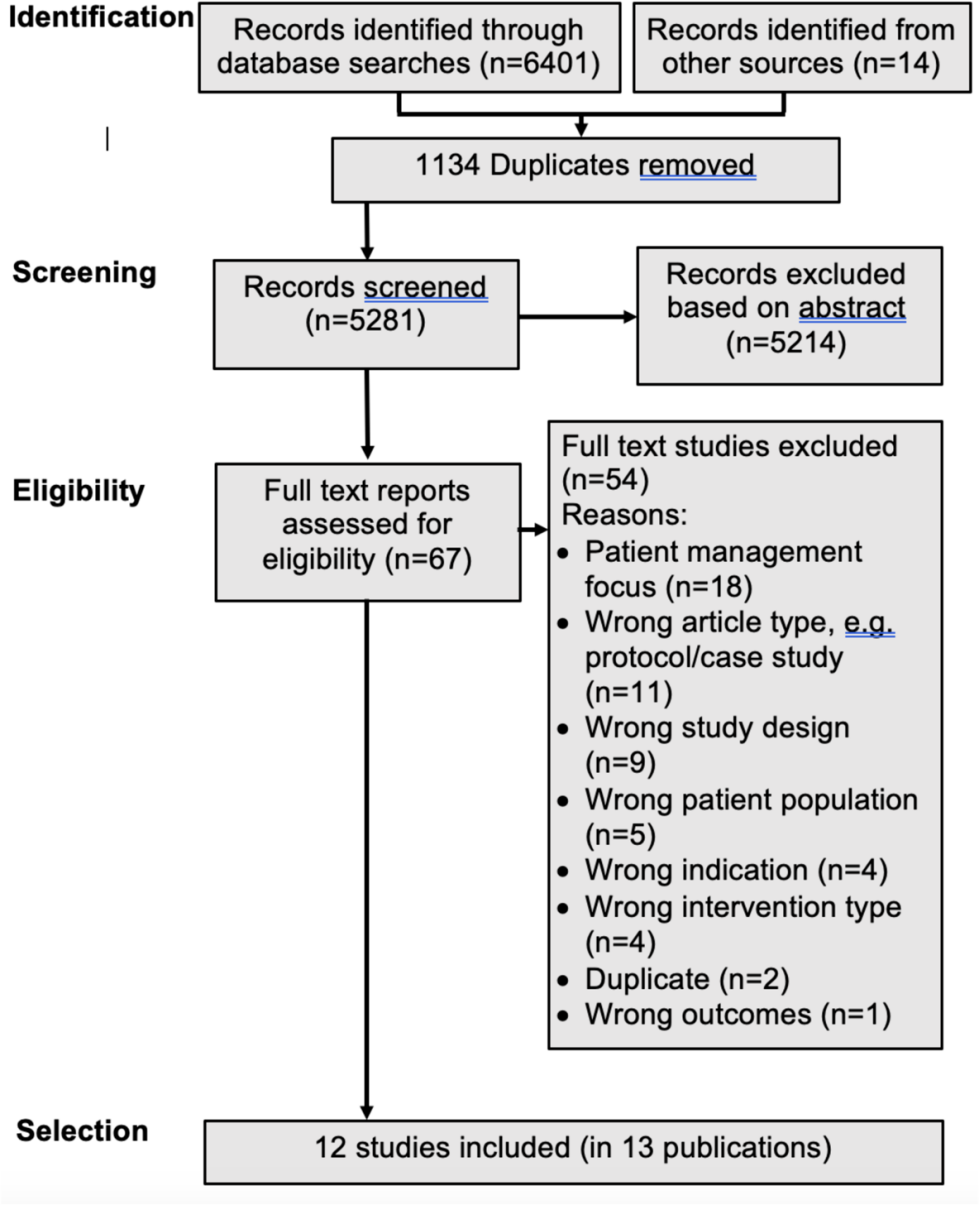
Flowchart of screening and selection process

In total, 6401 records were identified via database searches. With duplicates removed, 5281 were screened, with 5214 excluded after title and abstract screening. Fourteen records were identified from other sources including hand searching of study reference lists (backward citations), citing literature (forward citations), and trial databases. After a full-text review of the remaining articles, 12 studies (in 13 publications) were included (see Fig. 1: Flowchart). An inter-rater reliability analysis was conducted using Fleiss Kappa, an adaptation of Cohen’s Kappa utilised with 3 or more raters [27]. The analysis reflected moderate agreement for title and abstract screening (*κ* = 0.502) and substantial agreement for full-text screening (*κ* = 0.801) [28].

### Risk of bias and quality assessment

**Fig. 2:**
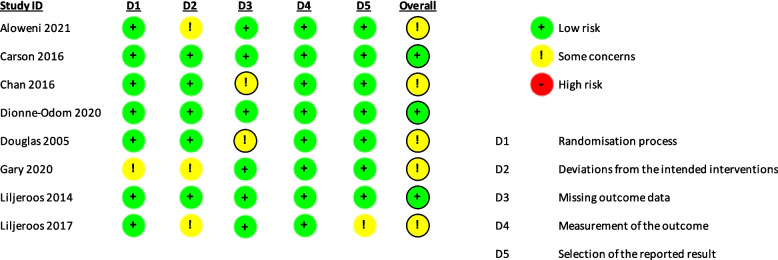
Cochrane RoB 2.0: summary of bias for each study (RCT)

**Fig. 3:**
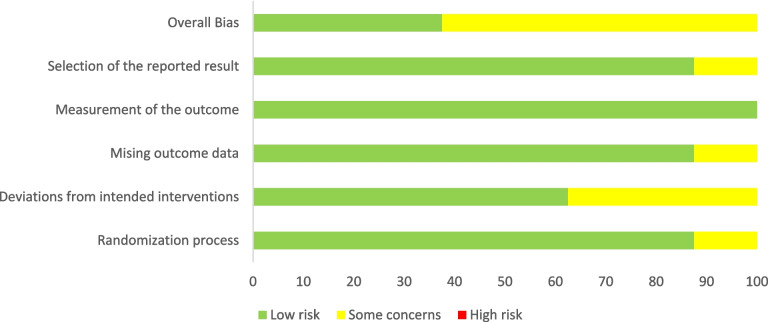
Cochrane RoB 2.0: summary of bias across all included RCTs

Results from the RoB 2.0 assessment are reported in Figs. 2 and 3. From these figures, it can be seen that of the 8 RCTs included in this review, 3 studies had an overall low risk of bias [29–31], whilst the remaining 5 had an overall unclear risk of bias, noted as ‘some concerns’ [32–35]. Figure 3 shows in which domains the concerns arose.

The remaining 5 studies were assessed using the Joanna Briggs Institute reviewer manual [25]. In 2 of the quasi-experimental studies, all or nearly all of the appraised domains are endorsed positively [36, 37]. The remaining 3 studies have positive endorsements of many of the applicable domains [38–40]. The results of this assessment are presented in Table 1.

**Table 1:** Risk of bias assessment for experimental studies

Study	1	2	3	4	5	6	7	8	9
Allen, 2008 [36]	Y	Y	Y	Y	Y	Y	Y	Y	Y
Bakitas 2017 [38]	Y	NA	NA	N	Y	U	Y	Y	Y
Hener, 1996 [37]	Y	Y	Y	Y	Y	Y	Y	U	Y
Law 2021 [39]	Y	NA	NA	N	Y	Y	Y	Y	Y
Sebern, 2012 [40]	Y	NA	NA	N	Y	U	Y	Y	Y

### Study characteristics

A total of 12 studies (13 publications) were included. Seven studies took place in the USA [29, 30, 34–36, 38, 40], 1 in Sweden (in 2 publications: [31, 41], 1 in Singapore [32], 2 in Hong Kong [33, 39], and 1 in Israel [37]. The majority of the included studies were RCTs [29–35, 41].

The overall sample comprised 1353 caregivers (Table 2). Study sample sizes ranged from 10 to 365 caregivers. The mean caregiver age ranged from 44 to 69.5 years. In all studies, a much higher proportion of caregivers were female (56–97%). Included studies reported on interventions for carers of those with a range of end-stage or advanced, non-malignant chronic conditions, including the following: heart failure [30, 31, 35, 38, 40, 41], renal failure [32, 33, 37], critical chronic illness [29, 34], and mixed chronic illness [36, 39].

**Table 2:** Study characteristics, population, study design, intervention delivery, structure, type, and follow-up

**Study (1st author, year, *location*)**	**Population** ***a. Condition(s); b. N(%); c. Age M* ± *SD; d. sex***	**Study design**	**Intervention delivery** ***a. Setting; b. Facilitator;*** ***c. Format; d. Recipient***	**Intervention structure** ***a. Intervention duration; b. No. of sessions; c. Frequency; d. Length of session***	**Intervention type (targeting)** ***a. Type; b. Targeting***	**Intervention follow-up**
Allen, 2008, [36] *USA*	Patient and family caregiver dyads (*n* = 31) *a. Advanced mixed chronic illness* *Intervention group (IG)* *b. n* = *17 dyads (55%)* *c. Cs: 57.8* ± *10.4; Pts: 75.4* ± *11.3 years* *d. Female Cs: 15 (88%); female Pts: 12 (71%)* *Control group (CG)* *b. n* = *14 dyads (45%)* *c. Cs: 55.1* ± *15.2; Pts: 75.3* ± *10.5 years* *d. Female Cs: 11 (79%); female Pts: 11 (79%)*	2-arm parallel group (intervention/ control) Randomised contact control group design	*a. Home* *b. Interventionists who received intensive training, observed by a licensed clinical psychologist* *c. Face-to-face* *d. Dyad: patient and caregiver (together)*	*a. Approximately 3 weeks* *b. 3 sessions* *c. Weekly (approx.)* *d. Session 1 M* = *82 min* *Session 2 M* = *66 min* *Session 3 M* = *70 min*	*a. Treatment components from life review and CBT* *b. Caregiver stress; depression; psychological wellbeing*	Post intervention (on study completion)
Aloweni, 2022, [32] *Singapore*	Family caregivers (*n* = 44) *a. Advanced renal disease (CKD)* *Intervention group (IG)* *b. n* = *16 (36%)* *c. 44.1* ± *10.3 years* *d. Female: 10 (63%)* *Control group (CG)* *b. n* = *28 (64%)* *c. 48.4* ± *15.2 years* *d. Female: 21 (75%)*	2-arm parallel group (intervention/ control) feasibility RCT	*a. Hospital and home* *b. A clinical psychologist trained in mindfulness* *c. Face-to-face* *d. Caregiver (family caregiver)*	*a. 4 days Mindfulness training (MT)* *b. 4 sessions* *c. Daily, with telephone contact every week for 4 weeks and monthly for 6 months to check on daily mindfulness practice* *d. 1 h*	*a. Third-wave cognitive-behavioural therapy: Mindfulness Therapy* *b. Caregiver stress; anxiety; HRQoL*	4 weeks, 3 months, 6 months
Bakitas 2017, [38] *USA*	Patient and caregiver dyads *a. Advanced heart failure* *Intervention group* *b. n* = *48 (100%)* *c. 64.9* ± *9.3 years* *d. Female, 39 (81.3%)* *No control group (CG)*	1 group feasibility study (of the ENABLE CHF-PC intervention trialled in Dionne-Odom, 2020, USA)	*a. Home/telephone* *b. Nurse-led/delivered—Five nurse coaches received 20 h of training including self-study of intervention protocols/ scripts and interactive role-play of 10 digitally recorded practice sessions. Nurse coaches were debriefed on their training sessions by the PI and Co-I who provided constructive feedback* *c. Telephone; manualised intervention* *d. Caregiver and patient*	*a. 24 weeks* *b. 4 sessions for caregivers* *c. Weekly; with monthly follow-up sessions; using ‘Charting Your Course’, educational guidebook* *d. M* = *46 min for weekly sessions*	*a. Structured, manualised; theory-based intervention* *b. Caregiver burden, anxiety, depression, QoL*	24 weeks
Carson, 2016, [29] *USA*	Family caregivers/Surrogate decision makers (*n* = 365) *a. Chronic critical illness* *Intervention group (IG)* *b. n* = *184 (50%)* *c. 51* ± *NR years* *d. Female, 128 (70%)* *Control group (CG)* *b. n* = *181 (50%)* *c. 51* ± *NR years* *d. Female: 131 (72%)*	2-arm parallel group (intervention/ control) RCT	*a. Hospital* *b. A palliative care physician and nurse practitioner (could include social workers, chaplains, or other disciplines as needed) with training in study approach—protocol guided* *c. Face-to-face* *d. Families/main caregiver*	*a. 10 days* *b. Minimum of 2 protocolised, interdisciplinary, informational support meetings/sessions* *c. Sessions 1 and 2 separated by 10 days* *d. NR*	*a. Information and support* *b. Anxiety; depression; PTSD symptomology*	3 months
Chan, 2016, [33] *Hong Kong*	Patient and family caregiver dyads (*n* = 29) *a. Advanced renal disease (CM)* *Intervention group (IG)* *b. n* = *14 dyads (48%)* *c. NR* *d. Female Cs: 11 (79%); female Pts, 6 (43%)* *Control group (CG)* *b. n* = *15 dyads (52%)* *c. NR* *d. Female Cs: 11 (73%); female Pts: 8 (53%)*	2-arm parallel group (intervention/ control) pilot RCT	*a. In clinic; home and telephone at follow-up* *b. Nurse, social worker, physician(renal)* *c. Face-to-face, with home visits and telephone at follow-up* *d. Caregiver and patient*	*a. 24 weeks* *b. 6–12 sessions* *c. 1–2 per month* *d. 30 min*	*a. Enhanced psychosocial support programme* *b. Caregiver burden, anxiety, depression*	1,3,6 months
Dionne-Odom, 2020, [43] *USA*	Caregivers (*n* = 158) *a. Advanced heart failure* *Intervention group (IG)* *b. n* = *82 (52%);* *c. 59.2* ± *12.4 years* *d. Female: 73 (89%)* *Control group (CG)* *b. n* = *76 (48%)* *c. 56.7* ± *10.8 years* *d. Female: 62 (82%)*	2-arm parallel group (intervention/ control) RCT	*a. Home* *b. Nurse-led/delivered — Four registered nurse coaches underwent 28 h of structured orientation and training overseen by the principal investigator, caregiving expert co-investigator, and study staff,* *c. Telephone; manualised intervention* *d. Caregivers*	*a. 48 weeks* *b. 4 sessions; monthly follow-up up to 48 weeks* *c. Weekly; monthly follow-up up to 48 months* *d. 20–60 min per session (M* = *44.1 min)*	*a. Manualised psychosocial and problem-solving support; theory-based (chronic care model) intervention* *b. Caregiver burden, anxiety, depression, QoL*	8, 16 weeks
Douglas, 2005, [34] *USA*	Family caregivers (*n* = 290) *a. Chronic critical illness* *Intervention group (IG)* *b. n* = *211 (73%)* *c. 53.1* ± *14.5 years* *d. Female, 156 (74%)* *Control group (CG)* *b. n* = *79 (27%)* *c. 52.6* ± *17.7 years* *d. Female: 54 (68%)*	2-arm parallel group (intervention/ control) RCT	*a. Hospital or telephone if living* > *30 miles from hospital site* *b. Advanced nurse practitioner led* *c. Face-to-face or by telephone depending on locations* *d. Patients and caregivers*	*a. 8 weeks* *b. Minimum of 8 APN-initiated contacts that constituted the intervention. Additional contacts were initiated by the patient, caregiver, health-care professional, or APN* *c. The median number of contacts during the 8 week study period was 30 for those in the experimental group* *d. NR*	*a. Structured intervention: coping/instrumental/emotional/social support* *b. Caregiver burden, depression, HRQoL*	2 months
Gary, 2020, [35] *USA*	Family caregivers (*n* = 127) *a. Advanced heart failure* *IG1: Psycho education (PE)* *b. n* = *44 (35%)* *c. 55* ± *11 years* *d. Female: 41 (93%)* *IG2: Psychoeducation* + *exercise (PE* + *E)* *b. n* = *48 (38%)* *c. 54* ± *10 years* *d. Female, 42 (88%)* *Control group (CG)* *b. n* = *35 (28%)* *c. 57* ± *14 years* *d. Female: 34 (97%)*	3-arm parallel group (2 interventions/ 1 control) RCT	*a. PE: Unclear; PE* + *E: exercise (E) aspect of intervention is home based* *b. NR* *c. PE: Educational group sessions* *PE* + *E: PE and an individualised exercise programme* *d. Caregivers*	*a. 24 weeks* *b. PE: 4 sessions; with follow-up phone calls weekly for first 12 weeks and then 2/month for the (next) 12-week maintenance period* *PE* + *E: 4 PE sessions; E: Approx. 36 sessions over first 12 weeks; NR 12-week maintenance period* *c. PE: Weekly* *PE* + *E: E Aerobic:3 times/week* *E Resistance: 2–3 times/week* *d. PE: NR* PE + E: E: *Aerobic: 30 min; resistance: 1–1.5 h*	*a. Psychoeducation involving active learning, group exercises and discussion, and coaching* *b. Caregiver strain*	6 months
Hener, 1996, [37] *Israel*	Patient and spouse caregiver dyads (*n* = 60) *a. Advanced renal disease (ESRD)* *IG1: Supportive (S)* *b. n* = *18 (30%)* *c. Cs: 50.8* ± *12.8 years* *d. Female Cs: 12 (67%); female Pts: 6 (33%)* *IG2: Cognitive-behavioural therapy (CBT)* *b. n* = *18 (30%)* *c. Cs, 53.1* ± *10.7 years* *d. Female Cs, 12 (67%); female Pts, 6 (33%)* *Control group (CG)* *b. n* = *24 (40%)* *c. Cs: 55.7* ± *10.0 years* *d. Female Cs: 16 (67%); female Pts: 8 (33%)*	3-arm parallel group (2 interventions/ 1 control) quasi-experimental study	*a. Home* *b. 2 clinical psychologists and 1 clinical social worker. Each therapist had 9–15 years’ experience in the specific treatment approach and both theoretical and clinical experience in family and short-term therapy* *c. Face-to-face* *d. Patient and caregiver (spouse)*	*a. 8 weeks* *b. 8 sessions* *c. 8 sessions in 8 weeks — possibly weekly, but unclear* *d. Approx. 80 min*	*a. Psychosocial and problem-solving support; theory-based (chronic care model) intervention* *b. Anxiety; depression; self-efficacy; distress*	Post intervention (end of programme)
Law, 2021, [39] *Hong Kong*	Patients (*n* = 74) and caregivers (*n* = 36) *a. End-stage, non-malignant, chronic diseases, including:* *Respiratory (55.4%)* *Renal failure (13.5%)* *b. n* = *36 (100%)* *c. Cs: 58.5* ± *16.1 years* *d. Female Cs: 31 (86%);* *female Pts: 25 (34%)*	Single-group pre-post comparison study	*a. Community* *b. 2 social workers (case managers), 1 nurse, 1 programme assistant, 3 professional volunteers (retired nurses), and 43 trained community volunteers. Community volunteers had* ≥ *20 h training before engaging in the programme* *c. Face-to-face, supplemented with phone calls* *d. Patients and caregivers*	*a. 3–4 months (active)* *b. 6–8 sessions* *c. Unclear* *d. Unclear*	*a. Education with psychosocial–spiritual support including stress management and creative therapeutic techniques* *b. Caregiver strain, psychological wellbeing*	Post active intervention (3 months)
Liljeroos, 2015, [31] *Sweden*	Patient and partner caregiver dyads (*n* = 155) *a. Advanced heart failure* *Intervention group (IG)* *b. n* = *71 (46%);* *c. Cs: 67.1* ± *12.1; Pts: 69.4* ± *13.6* *d. Female Cs: 49 (69%); female Pts: 22 (31%)* *Control group (CG)* *b. n* = *84 (54%)* *c. Cs: 69.5* ± *10.5; Pts: 72.9* ± *10.1* *d. Female Cs: 68 (81%); female Pts: 16 (19%)*	2-arm parallel group (intervention/ control) RCT	*a. Home/heart failure clinic* *b. Nurse-led; with computer-based programme and written materials. Nurses were experienced HF nurses who received three days of theoretical training followed by individual and practical training on how to perform the intervention* *c. Face-to-face* *d. Patient and caregiver (spouse)*	*a. 12 weeks* *b. 3 sessions* *c. At 2, 6, and 12 weeks after hospital discharge* *d. 60 min*	*a. Educational and psychosocial* *b. Depression, HRQoL*	24 months
Liljeroos, 2017, [41] *Sweden*	The same group as Liljeroos, 2014, focus on carer outcomes only (*n* = 155 dyads) Partner caregiver *a. Advanced heart failure* *Intervention Group (IG)* *b. n* = *71 (46%)* *c. Cs: 67.1* ± *12.1* *d. Female 49 (69%)* *Control group (CG)* *b. n* = *84 (54%)* *c. Cs: 69.5* ± *10.5* *d. Female 68 (81%)*	2-arm parallel group (intervention/ control) RCT	*a. Heart failure clinic* *b. Nurse-led; with computer-based programme and written materials. Nurses were experienced HF nurses who received 3 days of theoretical training followed by individual and practical training on how to perform the intervention* *c. Face-to-face* *d. Patient and caregiver (spouse)*	*a. 12 weeks* *b. 3 sessions* *c. At 2, 6, and 12 weeks after hospital discharge* *d. 60 min*	*a. Educational and psychosocial* *b. Caregiver strain*	24 months
Sebern, 2012, [40] *USA*	Patient and family caregiver dyads (*n* = 9) *a. Advanced heart failure* *Intervention group (IG)* *b. n* = *9 dyads (19 participants) (100%)* *c. Cs: 61* ± *19; Pts: 80* ± *9.5* *d. Female Cs: 10 (100%); female Pts: 5 (56%)* *No control froup*	1 group quasi-experimental design	*a. Home* *b. PhD and master’s-prepared nurses with clinical background in the management of HF* *c. Face-to-face — one-on-one and dyadic intervention for care partners managing HF. Care partners were also given copies of all worksheets and educational* *materials used with the SCDI* *d. Patient and caregiver*	*a. 12 weeks* *b. 7 sessions* *c. Weekly* *d. 60–120 min*	*a. Structured intervention* *b. Anxiety, depression, HRQoL*	Post intervention

*Cs* caregivers, *Pts* patients, *IG* intervention group, *CG* control group, *RCT* randomised controlled trial.

### Intervention characteristics

Interventions were delivered in the home [30, 35–40], hospital [29, 32, 34], or condition-specific clinic [31, 33, 41]. Interventions were described as delivered by the following: nurses, interventionists, or teams trained in the intervention approach [29–31, 36, 38, 41]; or professionals and volunteers with experience in the intervention type/method [32, 37, 39]. The remaining interventions reported no specific additional training in the intervention approach/methods but did report the involvement of chronic condition specialists [33–35, 40]. Intervention duration ranged from 4 days to 48 weeks, comprising 2–36 sessions. Where session duration was reported, sessions lasted from 30 min to 2 h (see Table 2).

### Control group

Three studies did not include a control/comparison group [38–40]. From the remaining studies, 7 reported the control group (CG) received ‘usual care’ [30–34, 37, 41]. Two studies reported the CG received usual care with additional informational materials [29, 35]. One study stated CG received minimal, non-specific support [36].

### Psychosocial outcomes for caregivers

All interventions measured changes in psychosocial outcomes (see Supplementary Materials: Table 2) in caregivers of those with advanced/end-stage chronic illness, including caregiver stress/burden/strain (*n* = 9: [30, 32–36, 38, 39, 41]); depression (*n* = 9: [29–31, 33, 34, 36–38, 40]); anxiety (*n* = 7: [29, 30, 32, 33, 37, 38, 40]); and quality of life (*n* = 7: [30–32, 34, 38, 40]). Two studies explored caregiver psychological wellbeing using a 3-item and single-item measure, respectively [36, 39], whilst another captured PTSD symptomology [29] and a further study measured caregiver distress [37].

### Overall changes

Significant improvements in at least one psychosocial outcome were reported in 9 of the studies [29, 31–33, 35–39]. These changes are reported below and described further in Table 3.

**Table 3:** Intervention description, outcomes, and results

Study (author, year, location)	Intervention description *a. Development; b. Description; c. Components*	Outcomes	Change in mean from baseline to follow-up	Significant difference *a. Follow-up (within)* *b. IG vs CG (between)*
Allen, 2008, [36] *USA*	*a. Developed within simplified version of existing stress process model. Legacy project comprises evidence-based aspects from life review and CBT; effective in reducing symptoms of depression. In this intervention, memories are elicited *via* questioning, and these shared memories are reflected through components of CBT (behavioural activation and homework)* *b. Intervention legacy activities included those to the following: (1) assist individuals or families in ‘life review’; (2) provide an output to be enjoyed by family/friends before/after patient death. Patients chose a Legacy project. The Legacy participant notebook (LPN) and Interventionist treatment manual (ITM) guided participants through development of their legacy project. The intervention comprised 3 in-home visits* *c. Session 1: Introduction of the LPN and problem-solving approach to help identify a Legacy project. Using standardised questions, the interventionist guided the dyad to discuss positive shared memories. The interventionist helped the dyad focus on a part of the patients’ life that could be represented in one tangible Legacy project (e.g. scrapbook, audiotape). They brainstormed potential means to portray the life story; then focussed on one project* *Session 2: Comprised interventionist coaching, reinforcing, and problem-solving as the dyad progress toward creating a tangible and lasting Legacy. Dyads were encouraged to use the Legacy materials in their daily lives* *Session 3: Comprised sharing their Legacy project with the interventionist and evaluating the intervention. Dyad was encouraged to construct other Legacies and share their work with family members and friends in their daily lives*	Caregiver Stress (CSS-R) Depression (CES-D) Psychological wellbeing (3 questions)	Baseline: 49.65 ± 7.35 T1: 48.94 ± 6.82 Baseline: 13.45 ± 8.38 T1: 12.58 ± 9.41 Baseline: 4.24 ± 1.03 T1: 4.18 ± 0.95	*a. − 0.71 (Ns)* *b. Significant difference found between groups (F(1, 29)* = *4.93, p* = *0.034) with increased caregiver stress evidenced in CG (2.93)* *a. − 0.87 (Ns)* *b. No significant differences between groups although a slight increase in depression in CG was noted (1.09)* *a. − 0.06 (Ns)* *b. No significant differences between groups although a slight improvement in wellbeing in CG was noted (0.3)*
Aloweni, 2022, [32] *Singapore*	*a. The Mindfulness training (MT) was developed by a clinical psychologist trained in mindfulness* *b. Intervention comprised 4 sessions of MT. At each session, the caregivers spent an hour learning and practising a mindfulness technique. At the end of each session, caregivers were instructed to practice the mindfulness exercises in the evening at home and provide feedback on their practice the next day. Caregivers were guided on daily practice during the sessions to ensure they could immerse themselves in the practice. Practicing breathing exercises in the morning and the body scan in the evening were recommended for caregivers. To foster compassion for themselves and their loved ones, caregivers were taught a loving-kindness and gratitude exercise. To facilitate the understanding and practice of MT, caregivers received reading and audio materials to guide home practice. A logbook was given to record their practice. To encourage participation, the research co-ordinator contacted the caregivers by phone each week for 4 weeks and then monthly for 6 months to check in on their daily practice* *c. Mindfulness training; practicing mindfulness techniques; guided practice; mindfulness exercises (home); reading and audio materials to guide practice for home use*	Caregiver stress (PSS) Anxiety (STAI-S) Anxiety (STAI-T) HRQoL (SF-36 PCS) HRQoL (SF-36 MCS)	Baseline: 17.81 ± 5.09 T1 (6m): Mean not reported Baseline: 40.31 ± 7.93 T1 (6m): M not reported Baseline: 39.0 ± 8.16 T1 (6m): M not reported Baseline: 42.14 ± NR T1 (6m): M not reported Baseline: 44.13 ± 9.97 T1 (6m): M not reported	*a. Significant—PSS scores significantly lower at 6 m* *b. No significant differences between groups. It was noted PSS IG scores were lower than CG (b* = *− 1.92, p* = *0.081)* *a. Ns* *b. No significant differences between groups. It was noted STAI-S scores in the IG were lower than those in the CG (b* = *− 2.16, p* = *0.311)* *a. Significant – STAI-T scores significantly lower at 6m* *b. No significant differences between groups. It was noted STAI-T IG scores were lower than CG (b* = *− 2.10, p* = *0.086)* *a. Ns* *b. No significant differences between groups. It was noted SF-36 PCS IG scores were lower than CG (b* = *− 0.77, p* = *0.084)* *a. Ns* *b. No significant differences found between groups although it was noted SF-36 MCS scores in the IG were higher than those in the CG (b* = *4.15, p* = *0.108)*
Bakitas 2017, [38] *USA*	*a. Informed by a proof-of-concept, formative evaluation study, which translated materials and protocols from a successful EPC ENABLE oncology model to a HF population* *b. Intervention (ENABLE CHF-PC) used in this study included the following: (1) an in-person outpatient palliative care consultation for patient (caregiver invited to attend) following National Consensus Guidelines; (2) weekly, semi-structured palliative care nurse coach (patients, 6 sessions; caregivers, 4 sessions) telephone and monthly follow-up sessions using ‘Charting Your Course’, an educational guidebook* *Sessions, conducted weekly, covered the following topics problem solving, self-care, symptom management, decision-making and advance care planning, and life review (patients only) that were tailored to individual participant needs. The life review sessions were based on the Outlook intervention (Steinhauser *et al*.)* *The goal of the sessions was to encourage participants to feel empowered and to develop skills that would assist them to make value-driven decisions about their medical and life-sustaining treatment choices as their disease worsened: Patients and caregivers were assigned separate nurse coaches to increase their sense of confidentiality* *c. Four carer sessions: Session 1: Problem solving; COPE Attitude; 2: Self-care; 3. Symptom management; 4: Core values, Talking about what matters most, Making decisions for the future*	Caregiver burden (MBCB — Total) (MBCB — Objective burden) (MBCB — Demand burden) (MBCB — Stress burden) Anxiety (HADS-A) Depression (HADS-D) QoL (BCOS)	Baseline: NR T1(24Wks): mean difference (MD) from BL-24Wks: − 3.1 (SE1.0) Baseline: NR T1(24Wks): MD from BL-24Wks: − 1.1 (0.5) Baseline: NR T1(24Wks): MD from BL-24Wks: − 0.6 (0.4) Baseline: NR T1(24Wks): MD from BL-24Wks: − 1.3 (0.4) Baseline: NR T1(24Wks): MD from BL-24Wks: − 0.2 (0.5) Baseline: NR T1(24Wks): MD from BL-24Wks: − 1.3 (0.7) Baseline: NR. T1(24 Wks): MD from BL-24Wks: 3.70 (2.0)	*a. Significant decrease in caregiver burden, mean difference* = *− 3.1 p* = *0.002* *b. No CG* *MBCB comprises 3 subscales:* *a. Significant decrease in caregiver (objective) burden, mean difference (MD)* = *− 1.1 p* = *0.02* *b. No CG* *a. Non-significant decrease in caregiver (demand) burden, MD* = *− 0.6, p* = *0.09* *b. No CG* *a. Significant decrease in caregiver (objective) burden, MD* = *− 1.3 p* = *0.001* *b. No CG* *a. Non-significant decrease in anxiety, MD* = − *0.2, p* = *0.69* *b. No CG* *a. Non-significant decrease in depression, MD* = − *1.3, p* = *0.08* *b. No CG* *a. Non-significant increase in QoL, MD* = *3.7, p* = *0.07* *b. No CG*
Carson, 2016, [29] *USA*	*a. The intervention meetings were structured according to a set of objectives and recommended topics (informed by literature). Protocol led* *b. A validated and widely available brochure describing chronic critical illness provided to caregivers. Research coordinators scheduled a minimum of 2 meetings with the support and information team comprising: a palliative care physician and nurse practitioner (could include social workers, chaplains, or others as needed)* *The first and second support and information team meetings targeted 2 key time points. The first meeting was conducted after 7 days of mechanical ventilation at the onset of chronic critical illness. The second meeting was conducted after further treatment was provided. Support and information team clinicians followed the main objectives of the meeting templates in the protocol but were allowed some flexibility for adapting the content of the meetings to the particular needs of each family* *c. Enhanced understanding of CCI; Family expectations; Long-term care; Values of patient and carer; Support and input as needed by family*	Anxiety (HADS-A) Depression (HADS-D) PTSD symptomology (IES-R)	Baseline: 9.5 ± 4.8 T1 (3m): 12.2 ± NR Baseline: 4.24 ± 1.03 T1: 4.18 ± 0.95 Baseline: NR T1: 25.9 ± NR	*Only between groups reported* *b. No significant differences in the HADS-A score at 3 months between the IG (M* = *7.2) and CG (M* = *6.4) p* = *0.09* *b. No significant differences in the HADS-D score at 3 months between the IG (M* = *5.0) and CG (M* = *5.0) p* = *0.93* *b. Significant differences in the PTSD score at 3 months between the IG (M* = *25.9) and CG (M* = *21.3) p* = *0.0495. Appears the support and information protocol-based intervention may have increased PTSD symptomology in caregivers*
Chan, 2016, [33] *Hong Kong*	*a. An enhanced psychosocial support programme was ‘put forward’ by a collaborative renal palliative care service in Hong Kong. Evidence-based intervention informed by existing research on published information regarding families’ needs in both end-stage renal disease and palliative care. No underpinning theory listed* *b. Caregivers received enhanced psychosocial support, i.e. education and intervention from an on-site palliative care nurse and designated social worker. The intervention adopted a proactive, comprehensive, multidisciplinary approach for patients and caregivers. The intervention consisted of 30-min sessions held once to twice monthly on the day of a patient’s joint clinic follow-up with a nurse, social worker, and physician. The palliative care nurse and social worker assessed each patient/caregiver pair before physician consultation and on the same day of the patient clinic appointment for the sake of caregiver convenience. The beginning of the first session was a needs assessment session. After the needs assessment, caregivers were given appropriate counselling and information accordingly. The psychosocial interventions were given based on individual needs. Home visits and telephone follow-ups were provided by a palliative care team in the intervention group* *c. Enhanced psychosocial support included counselling and psychosocial interventions by an on-site palliative care nurse and designated social worker*	Caregiver Burden (ZBI) Anxiety (HADS-A) Depression (HADS-D)	Baseline: 32.8 ± 12.2 T1(3M): 21.3 ± 6.6 T1(6M): 24.3 ± 6.3 Baseline: 9.9 ± 3.3 T1(3M): 6.5 ± 4.5 T1(6M): 8.5 ± 1.9 Baseline: 5.4 ± 4.5 T1(3M): 3.8 ± 3.1 T1(6M): 4.5 ± 1.9	*a. Significant decrease reported in caregiver burden at 3 months (p* = *0.02); however, whilst a decrease was still evident at 6 months, this was no longer significant (p* = *0.07)* *b. Significant differences in the caregiver burden at 3 months between the IG (M* = *21.3) and CG (M* = *33.4) p* = *0.001. This difference has decreased at 6 months, and although caregiver burden remains lower in the IG (24.3) vs CG (31.6), this difference is no longer significant (p* = *0.2)* *a. NR — decrease reported in anxiety at 3 months; whilst an increase in anxiety from 3–6 months was noted; this remained lower than the anxiety score at BL. No information provided on significance* *b. Significant differences in the anxiety at 3 months between the IG (M* = *6.5) and CG (M* = *11.0) p* = *0.03. This difference has decreased at 6 months, and although anxiety remains lower in the IG (8.5) vs CG (10.6), this difference is no longer significant (p* = *0.1)* *a. NR — decrease reported in depression at 3 months; whilst an increase in depression from 3–6 months was noted; this remained lower than the depression score at BL. No information provided on significance* *b. Differences in the depression between the IG (M* = *3.8) and CG (M* = *6.7) at 3 months, and 6 months IG (M* = *4.5) and CG (M* = *6.7). This difference has decreased at 6 months, and although anxiety remains lower in the IG (8.5) vs CG (7.4), these differences were non-significant (p* = *0.08 and p* = *0.01, respectively)*
Dionne-Odom, 2020, [30] *USA*	*a. Formative evaluation work was undertaken to adapt the ENABLE caregiver intervention from cancer to heart failure and refine the intervention *via* 2 single-group pilot trials (ENABLE CHF-PC)* *b. The nurse coach uses the manualized curriculum: ‘Charting Your Course (CYC): An Intervention for Patients with Heart Failure and their Families’ Nurse coaches paired with intervention-group family caregivers facilitated a series of phone sessions guided by a Charting Your Course Caregiver guidebook. Guidebooks were mailed to participants prior to their first session. Participants were encouraged to review session material prior to appointments with their nurse coach* *c. 4-session caregiver curriculum followed by monthly phone-based supportive care for 48 weeks or patient death* *Session 1: Introducing and defining palliative care, eliciting the caregiver’s illness understanding and the activities they do to support their care recipient, discussing problem solving using the COPE framework, and outlined steps of problem solving* *Session 2: review of self-care topics, relaxation techniques, how to ask for help, and identifying and building supports* *Session 3: partnering in symptom management; common physical and emotional symptoms in heart failure; and spirituality* *Session 4: values and the family member in patient decision-making, advance care planning, and decisions*	Caregiver Burden (MBCB-Objective) Caregiver Burden (MBCB-Demand) Caregiver Burden (MBCB-Stress) Anxiety (HADS-A) Depression (HADS-D) QoL (BCOS)	Baseline: 20.0 (SE0.3) T1(16Wk): 20.2 (0.5) Baseline: 11.6 (0.2) T1(16Wk): 11.1 (0.4) Baseline: 12.2 (0.3) T1(16Wk): 11.7 (0.4) Baseline: 3.9 (0.3) T1(16Wk): 3.8 (0.5) Baseline: 4.7 (0.3) T1(16Wk): 4.5 (0.5) Baseline: 65.2 (1.3) T1(16Wk): 66.9 (2.1)	*a. NR* *b. No significant differences between IG (20.2) and CG (19.7) at 16 weeks* *a. NR* *b. No significant differences between IG (11.1) and CG (11.6) at 16 weeks* *a. NR* *b. No significant differences between IG (11.7) and CG (12.2) at 16 weeks* *a. NR* *b. No significant differences in anxiety between IG (3.8) and CG (4.2) at 16 weeks* *a. NR* *b. No significant differences in depression between IG (4.5) and CG (4.4) at 16 weeks* *a. NR* *b. No significant differences in depression between IG (66.9) and CG (63.9) at 16 weeks*
Douglas, 2005, [34] *USA*	*a. Evidence based — Informed by several intervention studies in the caregiving literature. ‘The use of coping and social/ emotional support had been supported by several intervention studies in the caregiving literature’* *b. Intervention was structured in order to provide emotional, as well as instrumental support, and provided individualised case management services from an advanced practice nurse (APN) who had access to a pulmonologist, geriatrician, and bioethicist for guidance and collaboration* *The APNs assessed both patient and caregiver needs for assistance and then, through an individualised plan of care, provided assistance that was needed. Typical APN activities included attending team meetings at extended care facilities, helping caregivers prepare for the patient’s eventual return home, providing emotional support for caregivers, counselling caregivers about end-of-life options, providing referrals for support (physical and/or emotional) to caregivers, coordinating services among multiple providers, arranging follow-up care from specialists, and monitoring the patient’s condition and medications. APNs often served as advocates for the patients and the caregivers, and made phone calls to physicians on behalf of the patients or caregivers in order to facilitate the treatment plan, answer questions, or expedite care* *c. Specifically, the 8-week intervention provided the following: 1. Emotional support through discussion, referrals, and reassurance; and 2. Instrumental support through care coordination, education, and communication*	Caregiver burden (CRA) Disrupted Schedule Finance Concerns Lack of family support Physical Health concerns Self-esteem Depression (CES-D) QoL SF-8 (PCS)	Baseline: NR T1(2M): 3.2 ± 0.90 Baseline: NR T1(2M): 2.5 ± 0.95 Baseline: NR T1(2M): 2.1 ± 0.82 Baseline: NR T1(2M): 2.3 ± 0.71 Baseline: NR T1(2M): 4.2 ± 0.47 Baseline: NR T1(2M): 12.3 ± 11.5 Baseline: 52.9 ± 7.7 T1(2M): 51.3 ± 9.4	*a. NR* *b. Caregiver burden was measured using 5 subscales on the CRA. No significant differences were found on any of the subscales when IG and CG were compared (p* = > *0.05)* *a. NR* *b. No significant differences in scores between IG (12.3) and CG (12.2) at 2 months* *a. No significant differences reported* *b. No significant differences were found on any of the subscales when IG and CG were compared (p* = *0.85)*
Gary, 2020, [35] *USA*	*a. Evidence based — Informed by existing research/interventions* *b. PE four consecutive weekly group sessions consisting of usual care plus the psychoeducational (PE) intervention. The goal of PE was to provide caregivers with the recommended self-care management guidelines. In addition, caregivers focused on communication and strategies that provided motivation, social support, coping skills, and accessing resources* *PE* + *E received PE sessions but also performed the combined aerobic and resistance exercise programme for 12 weeks followed by a 12-week maintenance period. Progressive low-to-moderate-intensity walking was used for the aerobic exercise component. Colour-coded Thera-cords were used for the resistance exercise component* *c. PE: 4 psychoeducation session with the recommended self-care management guidelines; focused on communication and strategies that provided motivation, social support, coping skills, and accessing resources* *PE* + *E: PE* + *aerobic and resistance exercise*	Caregiver strain (2 questions) QoL (BCOS)	Caregiver strain was only recorded at BL PE Baseline: 52 ± 15 T1(6M): 60 ± 15 PE + E Baseline: 55 ± 18 T1(6M): 68 ± 21	*a. NR* *b. NR* *a. PE: Significant improvement in QoL in IG from baseline to 6M (p* = *0.001)* *a. PE* + *E: Significant improvement in QoL in IG from baseline to 6M (p* < *0.001)* *b. There was a significant TimexGroup effect (p* = *0.008), highlighting significant improvements in QoL in the IGs when compared to no change in QoL in the CG*
Hener, 1996, [37] *Israel*	*a. Evidence- based: Theory-driven models were used to develop and test the interventions: a) the model of working through of mourning (Horowitz. 1982; Kubler-Ross, 1969; Wright, 1983) and (b) the cognitive-behavioural model (Cohen, Evans, Stokols, & Krantz, 1986; Lazarus, 1991; Lazarus & Folkman, 1984; Moos & Schaefer, 1984)* *b. Eight sessions of treatment (either supportive therapy or CBT) were provided to each couple in their home. Each session lasted approximately 80 min* *c. Supportive Therapy: emphasised working through the mourning process, acceptance of the illness, and loss of health and life expectancy, by use of encouragement, ventilation, and catharsis. Emotional expression and experience were encouraged with support provided. Insight was stressed for the psychological distress that was being experienced. Easing of the damaged self-image and encouragement for the development of self-potential was also emphasised. Therapists were trained to deal with problems from couples. The patient and his or her spousal caregiver were encouraged to find ways of solving problems without the therapist providing a solution. Problems that arose reflected: difficulties in accepting loss; expressions of negative emotions and thoughts; relating as a couple; the struggle for independence in a situation of dependence; and the uncertainty of the future* *CBT: aimed to help the patient and their spousal caregiver find equilibrium between the demands of the environment and their personal and social resources. This was done by providing them with a different understanding of their situation and teaching new skills for coping with some of the major problems they face. The overall goal was to increase perceived self-control & self-efficacy. CBT focused on four specific areas: emotional, cognitive, behavioural, and interpersonal* *Sessions were also devoted to teaching different forms of relaxation, controlling anger, coping: with sleep problems, worry and anxiety, bad moods; family communications, and problems of intimacy. The patient and his or her spouse were given written materials, encouraged to practice, and taught self-reinforcement for success*	Anxiety (Mixed scale: items from PAIS, BSI, MBHI) Depression (Mixed scale: items from PAIS, BDI) Self-efficacy Distress (social) (Based on PAIS)	Supp: Baseline: 41.3 ± NR T1(END): 30.6 ± NR CBT: Baseline: 39.8 ± NR T1(END): 28.2 ± NR Supp: Baseline: 51.2 ± NR T1(END): 41.8 ± NR CBT: Baseline: 50.7 ± NR T1(END): 42.7 ± NR Supp: Baseline: 52.5 ± NR T1(END): 57.5 ± NR CBT: Baseline: 52.3 ± NR T1(END): 59.2 ± NR Supp: Baseline: 56.9 ± NR T1(END): 42.3 ± NR CBT: Baseline: 62.8 ± NR T1(END): 56.9 ± NR	*a. Significant reduction in Supportive IG anxiety from BL to end of programme (p* < *0.01)* *a. Significant reduction in CBT IG anxiety from BL to end of programme (p* < *0.01)* *b. Significant differences in anxiety were found, with contrasts confirming significant reductions in IGs at end of programme when compared to CG (increase in anxiety) (p* = < *0.01). No significant differences were found in anxiety when Supportive IG vs CBT IG were compared* *a. Significant reduction in Supportive IG depression from BL to end of programme (p* < *0.01)* *a. Significant reduction in CBT IG depression from BL to end of programme (p* < *0.01)* *b. Significant differences in depression were found, with contrasts confirming significant reductions in IGs at end of programme when compared to CG (increase in depression) (p* = < *0.01). No significant differences were found in depression when Supportive IG vs CBT IG were compared* *a. Significant improvement in Supportive IG self-efficacy from BL to end of programme (p* < *0.01)* *a. Significant improvement in CBT IG self-efficacy from BL to end of programme (p* < *0.01)* *b. Significant differences in self-efficacy were found, with contrasts confirming significant improvements in IGs at end of programme when compared to CG (reduction in self-efficacy) (p* = < *0.01). No significant differences were found in self-efficacy when Supportive IG vs CBT IG were compared* *a. Significant reduction in Supportive IG social distress from BL to end of programme (p* < *0.01)* *a. Significant improvement in CBT IG social distress from BL to end of programme (p* < *0.01)* *b. Significant differences in social distress were found, with contrasts confirming significant reductions in IGs at end of programme when compared to CG (increase in social distress) (p* < *0.01). Significant differences were found in social distress when Supportive IG vs CBT IG were compared at end of programme with significantly greater reductions in social distress seen for supportive care at end of programme (p* < *0.01)*
Law, 2021, [39] *Hong Kong*	*a. Evidence based: The programme was based on an existing approach which provides an evidence-based, empowerment-focused framework for this intervention* *b. The LRP is delivered by social workers, a nurse, a programme assistant, professional and community volunteers, allowing for delivery of the intervention *via* a mix of skills in education, symptom management, and support. Following initial assessment, shared intervention goals are agreed with patients and caregivers. These are facilitated over 6–8 home visits (across 3–4 months), with telephone support. Following this (active phase), volunteers continue to link in with the family to provide telephone support* *c. Four core dimensions to the intervention: 1. Empowering patients and caregivers in holistic symptom management and education. 2. Stress management skills to reduce emotional distress, with psychosocial and spiritual support. Includes use of creative therapies to facilitate discussion and life review. 3: Family discussions on careplanning, caregiving issues, and preparing for death. 4: Identifying practical needs of caregivers/families and identification of appropriate supports*	Caregiver Strain (Modified Caregiver Strain Index: C-M-CSI) Psychological wellbeing (single-item measure)	Baseline: 11.7 ± 7.0 T1(3M): 9.9 ± 5.6 Baseline: 5.5 ± 2.2 T1(3M): 6.2 ± 1.6	*a. Significant reductions in Caregiver strain from BL to 3M follow-up (p* < *0.01)* *b. No CG* *Ns change in psychological wellbeing (improved mood) from BL to 3M follow-up* *b. No CG*
Liljeroos, 2015, [31] *Sweden*	*a. Theory based: The theoretical framework for the study was based on an existing health promotion model, focused on enhancing self-efficacy, which has been successfully used as an educational programme* *b. Educational and psychosocial intervention. Included psychosocial support to maintain/strengthen the dyads’ physical and mental functions and perceived control. The intervention was delivered in three modules through nurse-led face-to-face counselling, a computer-based programme and written materials. The sessions took place at 2, 6, and 12 weeks after hospital discharge. Each of the three modules contained cognitive, supportive and behavioral components and outcomes. All sessions included education on heart failure and development of problem-solving skills to assist the dyads in recognising/modifying factors that contribute to psychological and emotional distress. The intervention focused on changing thoughts and behaviours, as well as implementing strategies for self-care behaviours* *c. Session 1: Increase dyads’ knowledge of the disease and treatment, improve mental and physical functions, and introduce self-care behaviours* *Session 2: Increase knowledge of the rationale for lifestyle changes, assess patient need for support, modify and strengthen caregiver behaviour* *Session 3: Increase knowledge of heart failure care and outcomes. It was a reinforcement of the intervention, and included an assessment of outcomes on support, behaviour, and repeated computer-based education. This session also assessed the partner’s need for support and perceived caregiver burden, in order to find strategies to improve control and self-care behaviour, and plan for the future*	Depression (BDI) HRQoL (SF-36 PCS) HRQoL (SF-36 MCS)	Baseline: NR T1(24M): Mean difference from BL-24M: 0.66 ± 0.68 Baseline: NR T1(24M): Mean difference from BL-24M: − 2.67 ± 0.93 Baseline: NR T1(24M): Mean difference from BL-24M: 3.49 ± 1.10	*a. Ns — patient/partner dyad* *b. Ns — analyses did not show any significant differences in Depression between the IG and CG dyad outcomes* *a. Ns — patient/partner dyad* *b. Ns — analyses did not show any significant differences in HRQoL (PCS) between the patients in the IG and CG; however caregivers in the IG had a significantly greater decrease in HRQoL (PCS)* *(p* < *0.05) than the CG* *a. Ns — patient/partner dyad* *b. Ns — analyses did not show any significant differences in HRQoL (MCS) between the IG and CG dyad outcomes*
Liljeroos, 2017, [41] *Sweden*	*As above: Liljeroos, 2015, Sweden – Caregiver outcomes only*	Caregiver burden (CBS)	Baseline: 1.7 ± 0.5 T1(24M): Mean difference 0.10 ± 0.46	*a. NR separately for IG* *b. No significant difference in caregiver burden between IG and CG at 24M (p* = *0.803)* *Please note: CBS comprises 5 subscales; no significant between group differences on any of these subscales*
Sebern, 2012, [40] *USA*	*a. Theory (shared care) and evidence based. Relies on existing findings for similar studies; and adapts aspects of existing intervention for care partners of dementia patients; aspects of this intervention were adapted for HF* *b. The SCDI is a structured, one-to-one, and dyadic intervention for care partners managing HF. Each care partner dyad participated in seven sessions, which were conducted in either a joint or mixed format. In joint sessions, the interventionist and care partners met together for the entire time. Mixed-format sessions began and ended jointly, but also included time for separate meetings with the interventionist. Although the SCDI was a structured intervention, the interventionist could digress if unexpected needs arose* *c. Structured intervention comprising 7 sessions: 1. Understanding self-care in HF; 2. taking care of yourself—Taking care of each other; 3. Care Values and preferences; 4. Care preferences; 5. Family and Friends; 6. Community resources; 7. Looking to the future*	Anxiety (STAI) Depression (PHQ-9) HRQoL (SF-36: General) (SF-36: Physical) (SF-36: Emotional) (SF-36: Fatigue) (SF-36: Pain)	Baseline: 1.3 ± 0.34 T1(8Wk): 1.4 ± 0.35 Baseline: 2.1 ± 2.8 T1(8Wk): 2.2 ± 2.7 Baseline: 55.4 ± 17.8 T1(8Wk): 52.4 ± 19.7 Baseline: 77.5 ± 28.3 T1(8Wk): 78.0 ± 24.7 Baseline: 84.4 ± 13.9 T1(8Wk): 90.4 ± 9.2 Baseline: 57.5 ± 25.0 T1(8Wk): 70.5 ± 25.2 Baseline: 66.5 ± 20.2 T1(8Wk): 84.0 ± 18.7	*a. There was minimal change between baseline anxiety and Week 8 anxiety (d* = *0.15). Caregiver anxiety was low throughout the intervention (baseline M* = *1.3 and Week 8 M* = *1.4)* *b. No CG* *a. There was minimal change between baseline and Week 8 depression (d* = *0.04). Caregiver depression was low throughout the intervention (baseline M* = *2.1 and Week 8 M* = *2.8)* *b. No CG* *a. Data supported improved status for SF-36 subscales: (a) emotional wellbeing improved for 5 caregivers (d* = *0.51), (b) 9 had improvement in fatigue (d* = *0.52), and (c) 8 had improvement for pain (d* = *0.90)* *b. No CG*

### *Caregiver burden*

Of the 9 studies reporting on caregiver stress/burden/strain, 5 evidenced significant reductions in burden/strain [32, 33, 36, 38, 39], with 3 of these studies [32, 36, 38] evidencing significant reductions in caregiver burden within the intervention group (IG) across time. One study [39] had no CG but highlighted significant reduction in caregiver strain across time. The remaining study [33] evidenced significant differences between IG and CG, with significantly lower levels of caregiver burden reported in the IG at follow-up.

### Depression

Of the 9 studies reporting on depression, only one reported a significant reduction in depression within the IG across time, and also a significant difference in depression between IG and CG at follow-up, favouring the IG, i.e. the IG had significantly lower levels of depression when compared to the CG [37].

### Anxiety

Of the 7 studies reporting anxiety, two reported significant reductions in anxiety within the IG across time [32, 37]. Two studies reported significant differences in anxiety between IG and CG, with significantly lower levels of anxiety evidenced in the IG post intervention [33, 37].

### Quality of life (QoL)

Two of the 7 studies reporting on QoL reported significant changes among caregivers [31, 35]. One study reported significant improvement in IG QoL across time [35], with both studies reporting a significant improvement in QoL in the IG compared with the CG at follow-up [31, 35].

### Other psychosocial outcomes

One study [37] reported significant improvements in self-efficacy in the IG across time, but also significant differences in self-efficacy between the IG and CG, with higher levels of self-efficacy reported in the IG. Results were mirrored in relation to distress, with levels of IG distress significantly lower across time and levels of distress significantly lower among IG compared to CG at follow-up [37]. One study reported changes in PTSD, with significant differences found between IG and CG, with the intervention seemingly having a detrimental effect on PTSD, with IG levels significantly higher than those reported by CG post intervention [29].

### Intervention type

Details on intervention development, content, and components are reported in Table 3. Seven studies were described as psychosocial interventions [30, 32, 33, 36–39]. Four of these studies resulted in significant reduction in caregiver burden among IG participants [32, 33, 36, 38]. Law [39] reported significant reduction in caregiver strain among caregivers in their study across time. One study evidenced significant reduction in IG depression [37], with two psychosocial studies reporting significant reductions in IG anxiety [33, 37]. One psychosocial intervention reported significant improvement in self-efficacy, and significant reduction in distress, among IG caregivers [37].

Three interventions were psychoeducational in nature [31, 35, 41]. Significant improvements in IG QoL were noted in two psychoeducational interventions [31, 35].

The remaining three interventions were described as providing information, support, or structured provision for caregivers [29, 34, 40]. No significant changes were noted in psychosocial outcomes among participants in these studies.

### Intervention development

Four studies report development of the intervention from an existing evidence base, relying on findings around caregiver need, caregiver intervention, and effective approaches [33–35, 39]. Of these studies, two reported significant reduction in caregiver burden/strain [33, 39], with another reporting a significant improvement in QoL among the IG [35]. Three studies cite clear theoretical frameworks guiding intervention development [31, 32, 41]. Again, two studies report a significant reduction in caregiver burden [32, 39], with another reporting significant improvement in IG QoL [31].

Two further studies were highlighted as theory-based interventions, but these studies based their intervention on an existing oncology model and intervention adapted for an advanced chronic illness population and caregivers [30, 38]. One study reported a significant reduction in caregiver burden [38].

Two studies described intervention development guided by both theory and evidence [36, 37]. One study reported significant reductions in caregiver burden [36]. The second study highlighted significant reductions in depression, anxiety, and distress among the IG, with significant improvement noted in self-efficacy among IG also [37].

One study reports a theory and evidence-based intervention for caregivers, adapted from a dementia care intervention [40]. The remaining study provides little detail on intervention development [29]. No significant positive intervention effects were reported for psychosocial outcomes in either of these studies.

### Intervention delivery

Of the 7 studies involving patient and carer dyads [31, 33, 36–38, 41], five reported significant changes in psychosocial outcomes. Significant reductions in caregiver burden were noted in three of these studies [33, 36, 38]. Significant reductions in depression, anxiety, and distress, and significant improvements in self-efficacy, were recorded in the IG in one dyad study [37], whilst significant improvements in QoL were recorded in another [31]. Law [39], although not explicitly described as a dyad study, involves both patients and caregivers, with significant reductions in caregiver strain reported among caregivers.

The remaining studies [29, 30, 32, 34, 35] were aimed at caregivers only (Table 2). One study evidenced significant improvement in caregiver burden [32]. Another evidenced significant reduction in caregiver anxiety in the IG [32], with a further study reporting higher levels of QoL among the IG [35].

Outcomes associated with intervention delivery among the studies are mixed, with no clear conclusions able to be drawn in relation to intervention delivery methods, setting, duration, or frequency.

## Discussion

### Summary of findings

This review, to our knowledge, is the first to explore the effectiveness of interventions in improving psychosocial outcomes for caregivers of those with advanced, non-malignant, chronic conditions. The studies included in this review were rigorously assessed for risk of bias and quality. From the 8 RCTs in this review, 3 had an overall low risk of bias [29–31], whilst the remaining 5 had an overall unclear risk of bias, noted as ‘some concerns’ [32–35, 41]. The quality of all studies was assessed ahead of inclusion in the study. Given the quality of the included studies, we hope the findings will provide useful insight and guidance for future research in this area.

The aim of this review was to identify what interventions exist for caregivers of those living with advanced, non-malignant, chronic illness and to explore the efficacy of these. The review search strategy identified numerous records for screening and review, evidencing that a lot of research had been conducted to highlight the impact of caring on this cohort of carers via cross-sectional studies, and also to evidence their experiences via detailed qualitative work; however, this information has not been as readily translated into interventions for carers of those with advanced chronic illness [10, 17].

Twelve studies (*n* = 13 publications) fit the review inclusion criteria, despite the incidence of advanced chronic illness within our aging population. This perhaps supports the calls within the literature for improved provision [18] and the need for tailored, culturally appropriate, psychosocial interventions for this population [10, 17].

The findings highlight some trends, seemingly associated with improved psychosocial outcomes among caregivers for those with advanced chronic illness. Intervention delivery yielded mixed results; however, interventions reported in the review tended to most commonly be delivered face-to-face, at home. Given the involvement of dyads in these interventions (*n* = 6), delivery in the home may be reflective of preference of patients at end-of-life to be cared for, and to die, at home [42]. Although significant changes were reported for dyad interventions and interventions for carers only, dyad interventions were more readily associated with positive changes in outcomes for caregivers [31, 33, 36–38]. Perhaps unsurprisingly, psychosocial interventions were more likely to see positive changes in psychosocial outcomes for caregivers, with 6 psychosocial interventions evidencing improvements in caregiver outcomes including caregiver burden/strain, depression, anxiety, self-efficacy, and distress [32, 33, 36–39]. Psychoeducational interventions did not significantly improve psychosocial outcomes, except for QoL [31, 35].

Excluding those adapted from other interventions, some studies pointed to intervention development informed by evidence [33–35, 39], theory [31, 32, 41], or both [36, 37]. Although findings in relation to intervention development were mixed, the most effective intervention, in terms of the number of outcomes improved, was combined evidence based and theory driven [37].

Interventions adapted from other conditions, i.e. oncology and dementia [30, 38, 40] did not seem to translate the desired outcomes to the advanced chronic illness caregiver populations targeted, with only a significant within-group reduction in caregiver burden evidenced in one study [38]. It should be noted that the ENABLE CHF-PC intervention [30, 38] did undergo consultation to adapt to a new caregiver population, but translation was informed by the literature, expert consultation, and clinician input, with caregiver input only at the testing phase to assess acceptability and satisfaction [43]. Perhaps co-production of the adapted intervention, involving the target population of caregivers in the development and adaptation of the intervention, may have a greater impact [44].

The findings appear to support the case for interventions for this cohort of caregivers to be as follows: evidence-based, psychosocial, developed within an appropriate psychological framework, delivered at home, and involving the patient-carer dyad. However, it may be important to note that no explicit mention of caregiver involvement in the earliest stages of intervention development is outlined in the included studies. Given the growing body of literature in relation to co-production of caregiver interventions, this may be an important consideration which would improve the impact or effectiveness of interventions for caregiving populations [44, 45]. Interventions are likely to be most effective when targeted at the recognised needs of the caregiver population [42].

Another finding from the review recognises female caregivers outnumber males in all included studies, regardless of whether the patient being cared for is male or female. It may be important to consider gender-specific aspects in intervention development, e.g. around caregiver burden and coping styles [46, 47].

### Limitations

There are some limitations to the current literature. All studies rely on the use of self-report measures to capture caregiver data at different points in time; however, measures used in the studies differed, with a lack of consistency in the measures used, making comparison across outcomes more difficult. Future studies should carefully consider the measures used to capture caregiver outcomes to ensure reliability and validity of findings. Some studies struggled with small sample sizes, which may have implications for statistical power, and although a potential limitation, it is not unexpected in studies focused on those with advanced illness and their caregivers [48]. This is often compounded by caregivers’ lack of recognition of their caring role, as some do not identify as a carer, or do not consider their own needs and wellbeing [3].

Optimal intervention duration and frequency of sessions need further exploration, with results from this review unable to shed any significant light on these aspects. It may be important to also consider that the evidence on the effectiveness of interventions for caregivers in cancer has seen substantial growth within the literature over the last two decades [42]. It is hoped this pattern will be mirrored for caregiver interventions in chronic illness, with research in this area seemingly still in its infancy.

## Conclusion

The results of this review suggest that interventions for caregivers of those with advanced, non-malignant, chronic conditions can positively affect psychosocial outcomes among this population. However, the effects of these interventions are mixed, with some studies having greater impacts than others on caregiver burden, depression, anxiety, and quality of life. The interventions in this review vary in relation to design, delivery, duration, content, structure, and outcomes. It is clear information on interventions for caregivers for patients with advanced chronic illness is scant. Longitudinal studies, for example longer-term RCTs and observational studies, on intervention effectiveness over time, are needed to add to our understanding of efficacy and sustained impact. More sufficiently powered, robust, high-quality trials assessing the efficacy of interventions developed for use among this population are needed. Given the proposed growth in chronic illness, consideration should be given to increasing the reach and scalability of effective interventions for this cohort of caregivers, with online delivery offering an option for this. Given the isolation that can be experienced by caregivers, further research should explore the effectiveness of group interventions, as well as those targeting caregivers or dyads. With this in mind, this review suggests interventions developed for use and testing among caregivers of those with advanced, non-malignant, chronic illness should be as follows: evidence-based, developed within an appropriate theoretical framework, target both caregiver and patient dyad, delivered at home as these appear to hold more promise.

## Supplementary Information

Additional file 1: Supplementary materials.

## Data Availability

Search strategy can be found in supplemental materials.

## References

[1] Llop-Medina L, Fu Y, Garcés-Ferrer J, Doñate-Martínez A. Palliative care in older people with multimorbidities: a scoping review on the palliative care needs of patients, carers, and health professionals. Int J Environ Res Public Health. 2022;19(6):3195. 35328881 10.3390/ijerph19063195 PMC8954932

[2] World Health Organisation. Palliative care: key facts. 2020. Available from: https://www.who.int/news-room/fact-sheets/detail/palliative-care

[3] Carduff E, Finucane A, Kendall M, Jarvis A, Harrison N, Greenacre J, Murray SA. Understanding the barriers to identifying carers of people with advanced illness in primary care: triangulating three data sources. BMC Fam Pract. 2014;15(1):1–10. 24690099 10.1186/1471-2296-15-48 PMC3992158

[4] Drummond M, Johnston B, Quinn TJ. Measuring the success of interventions for caregivers: a focussed systematic review. Curr Opin Support Palliat Care. 2019;13(4):351. 31689272 10.1097/SPC.0000000000000461 PMC6867664

[5] Carers UK. Facts & Figures 2022. Available from: https://www.carersuk.org/news-and-campaigns/press-releases/facts-and-figures.

[6] Auclair I, Bourbonnais A, Lavoie A, Leclerc-Loiselle J. Protocol: inclusion of informal caregivers in the palliative and end-of-life care of older adults: a scoping review protocol. BMJ open. 2022;12(4):1–7. 10.1136/bmjopen-2021-053858 PMC9014003 35428622

[7] Wingham J, Frost J, Britten N, Greaves C, Abraham C, Warren FC, et al. Caregiver outcomes of the REACH-HF multicentre randomized controlled trial of home-based rehabilitation for heart failure with reduced ejection fraction. Eur J Cardiovasc Nurs. 2019;18(7):611–20. 31117815 10.1177/1474515119850011 PMC6764010

[8] Stephanou M. Caregiver burden: Support needed for those who support others and the National Health Service. Patient Exp J. 2023;10(2):23–33.

[9] World Health Organisation. WHO Definition of Palliative Care. 2007. Available from: http://www.who.int/cancer/palliative/definition/en/.

[10] Fusi-Schmidhauser T, Froggatt K, Preston N. Living with advanced chronic obstructive pulmonary disease: a qualitative interview study with patients and informal carers. J Obstruct Pulmon Dis. 2020;17(4):410–8. 10.1080/15412555.2020.1782867 32586144

[11] Carswell C, Yaqoob M, Gilbert P, Kuan Y, Laurente G, McGuigan K, et al., editors. Exploration of caregiver experiences of conservatively managed end-stage kidney disease to inform development of a psychosocial intervention: the ACORN study protocol. Healthcare. 2021;9(12)Article 1731:MDPI. 10.3390/healthcare9121731 PMC8701309 34946457

[12] Galvin M, Corr B, Madden C, Mays I, McQuillan R, Timonen V, et al. Caregiving in ALS–a mixed methods approach to the study of burden. BMC Palliat Care. 2016;15(1):1–12. 27596749 10.1186/s12904-016-0153-0 PMC5011853

[13] Wang T, Molassiotis A, Chung BPM, Tan J-Y. Unmet care needs of advanced cancer patients and their informal caregivers: a systematic review. BMC Palliat Care. 2018;17(1):1–29. 10.1186/s12904-018-0346-9 PMC6057056 30037346

[14] Kang X, Li Z, Nolan MT. Informal caregivers’ experiences of caring for patients with chronic heart failure: systematic review and metasynthesis of qualitative studies. J Cardiovasc Nurs. 2011;26(5):386. 21263337 10.1097/JCN.0b013e3182076a69 PMC8976444

[15] Bakas T, Pressler SJ, Johnson EA, Nauser JA, Shaneyfelt T. Family caregiving in heart failure. Nurs Res. 2006;55(3):180–8. 16708042 10.1097/00006199-200605000-00004

[16] Traue D, Ross J. Palliative care in non-malignant diseases. J R Soc Med. 2005;98(11):503–6. 16260799 10.1258/jrsm.98.11.503 PMC1275998

[17] Teixeira MJC, Abreu W, Costa N, Maddocks M. Understanding family caregivers’ needs to support relatives with advanced progressive disease at home: an ethnographic study in rural Portugal. BMC Palliat Care. 2020;19(1):1–11. 32450848 10.1186/s12904-020-00583-4 PMC7249372

[18] Marco DJT, Thomas K, Ivynian S, Wilding H, Parker D, Tieman J, Hudson P. Family carer needs in advanced disease: systematic review of reviews. BMJ Support Palliat Care. 2022;12(2):132–41. 34996834 10.1136/bmjspcare-2021-003299

[19] Motamedi M, Brandenburg C, Bakhit M, Michaleff ZA, Albarqouni L, Clark J, et al. Concerns and potential improvements in end-of-life care from the perspectives of older patients and informal caregivers: a scoping review. BMC Geriatr. 2021;21:1–12. 34930177 10.1186/s12877-021-02680-2 PMC8690959

[20] Lewis ET, Harrison R, Hanly L, Psirides A, Zammit A, McFarland K, et al. End-of-life priorities of older adults with terminal illness and caregivers: a qualitative consultation. Health Expect. 2019;22(3):405–14. 30614161 10.1111/hex.12860 PMC6543262

[21] Lindt N, van Berkel J, Mulder BC. Determinants of overburdening among informal carers: a systematic review. BMC Geriatr. 2020;20(1):1–12. 10.1186/s12877-020-01708-3 PMC7448315 32847493

[22] Moher D, Liberati A, Tetzlaff J, Altman DG, PRISMA Group. Preferred reporting items for systematic reviews and meta-analyses: the PRISMA statement. Ann Intern Med. 2009;151(4):264–9. 19622511 10.7326/0003-4819-151-4-200908180-00135

[23] Covidence. Covidence systematic review software Melbourne, Australia: Veritas Health Innovation; 2022. Available from: www.covidence.org.

[24] Sterne JA, Savović J, Page MJ, Elbers RG, Blencowe NS, Boutron I, et al. RoB 2: a revised tool for assessing risk of bias in randomised trials. bmj. 2019;366:1–8. 10.1136/bmj.l4898 31462531

[25] Tufanaru C, Munn Z, Aromataris E, Campbell J, Hopp L. Chapter 3: Systematic reviews of effectiveness. In: Aromataris E. MZ, editor. JBI Manual for Evidence Synthesis: JBI. 2020:72–134.

[26] Centre for Reviews and Dissemination. Systematic Reviews: CRD’s guidance for undertaking reviews in health care. York, England: York Publishing Services; 2008.

[27] McHugh ML. Interrater reliability: the kappa statistic. Biochem Med (Zagreb). 2012;22(3):276–82. 23092060 PMC3900052

[28] Landis JR, Koch GG. The Measurement of Observer Agreement for Categorical Data. Biometrics. 1977;33(1):159–74. 843571

[29] Carson SS, Cox CE, Wallenstein S, Hanson LC, Danis M, Tulsky JA, et al. Effect of palliative care–led meetings for families of patients with chronic critical illness: a randomized clinical trial. JAMA. 2016;316(1):51–62. 27380343 10.1001/jama.2016.8474 PMC5538801

[30] Dionne-Odom JN, Ejem DB, Wells R, Azuero A, Stockdill ML, Keebler K, et al. Effects of a telehealth early palliative care intervention for family caregivers of persons with advanced heart failure: the ENABLE CHF-PC randomized clinical trial. JAMA Net Open. 2020;3(4):e202583-e. 10.1001/jamanetworkopen.2020.2583 PMC7154802 32282044

[31] Liljeroos M, Ågren S, Jaarsma T, Årestedt K, Strömberg A. Long term follow-up after a randomized integrated educational and psychosocial intervention in patient-partner dyads affected by heart failure. PLoS ONE. 2015;10(9): e0138058. 26406475 10.1371/journal.pone.0138058 PMC4583392

[32] Aloweni F, Doshi K, Agus N, Fook-Chong S, Wu SY, Kong LP, et al. Evaluating the feasibility and effectiveness of a mindfulness-based intervention on stress and anxiety of family caregivers managing peritoneal dialysis. Proceedings of Singapore Healthcare. 2022;31:20101058211054910.

[33] Chan KY, Yip T, Yap DY, Sham MK, Wong YC, Lau VWK, et al. Enhanced psychosocial support for caregiver burden for patients with chronic kidney failure choosing not to be treated by dialysis or transplantation: a pilot randomized controlled trial. Am J Kidney Dis. 2016;67(4):585–92. 26549852 10.1053/j.ajkd.2015.09.021

[34] Douglas SL, Daly BJ, Kelley CG, O’Toole E, Montenegro H. Impact of a disease management program upon caregivers of chronically critically ill patients. Chest. 2005;128(6):3925–36. 16354865 10.1378/chest.128.6.3925

[35] Gary R, Dunbar SB, Higgins M, Butts B, Corwin E, Hepburn K, et al. An intervention to improve physical function and caregiver perceptions in family caregivers of persons with heart failure. J Appl Gerontol. 2020;39(2):181–91. 29347863 10.1177/0733464817746757 PMC6026574

[36] Allen RS, Hilgeman MM, Ege MA, Shuster JL Jr, Burgio LD. Legacy activities as interventions approaching the end of life. J Palliat Med. 2008;11(7):1029–38. 18788966 10.1089/jpm.2007.0294 PMC2664509

[37] Hener T, Matisyohu W, Har-Even D. Supportive versus cognitive–behavioral intervention programs in achieving adjustment to home peritoneal kidney dialysis. J Consult Clin Psychol. 1996;64(4):731. 8803363 10.1037//0022-006x.64.4.731

[38] Bakitas M, Dionne-Odom JN, Pamboukian SV, Tallaj J, Kvale E, Swetz KM, et al. Engaging patients and families to create a feasible clinical trial integrating palliative and heart failure care: results of the ENABLE CHF-PC pilot clinical trial. BMC Palliat Care. 2017;16(1):1–13. 28859648 10.1186/s12904-017-0226-8 PMC5580310

[39] Law MC, Lau BHP, Kwok AY, Lee JS, Lui RN, Liu K, et al. Empowering families facing end-stage nonmalignant chronic diseases with a holistic, transdisciplinary, community-based intervention: 3 months outcome of the Life Rainbow Program. Palliat Support Care. 2021;19(5):530–9. 33267934 10.1017/S1478951520001224

[40] Sebern MD, Woda A. Shared care dyadic intervention: outcome patterns for heart failure care partners. West J Nurs Res. 2012;34(3):289–316. 21383082 10.1177/0193945911399088

[41] Liljeroos M, Ågren S, Jaarsma T, Årestedt K, Strömberg A. Long-term effects of a dyadic psycho-educational intervention on caregiver burden and morbidity in partners of patients with heart failure: a randomized controlled trial. Qual Life Res. 2017;26:367–79. 27631892 10.1007/s11136-016-1400-9

[42] Ahn S, Romo RD, Campbell CL. A systematic review of interventions for family caregivers who care for patients with advanced cancer at home. Patient Educ Couns. 2020;103(8):1518–30. 32201172 10.1016/j.pec.2020.03.012 PMC7311285

[43] Dionne-Odom JN, Kono A, Frost J, Jackson L, Ellis D, Ahmed A, et al. Translating and testing the ENABLE: CHF-PC concurrent palliative care model for older adults with heart failure and their family caregivers. J Palliat Med. 2014;17(9):995–1004. 25072240 10.1089/jpm.2013.0680 PMC4158985

[44] Robert G, Donetto S, Williams O. Co-designing healthcare services with patients. In: Loeffler E, Bovaird T, editors. The palgrave handbook of co-production of public services and outcomes. London: Palgrave Macmillan; 2021. p. 313–33.

[45] Santin O, McShane T, Hudson P, Prue G. Using a six-step co-design model to develop and test a peer-led web-based resource (PLWR) to support informal carers of cancer patients. Psychooncology. 2019;28(3):518–24. 30597666 10.1002/pon.4969 PMC6590360

[46] Swinkels JC, Broese van Groenou MI, de Boer A, Tilburg TGv. Male and female partner-caregivers’ burden: does it get worse over time? Gerontologist. 2019;59(6):1103–11. 30321338 10.1093/geront/gny132

[47] Schrank B, Ebert-Vogel A, Amering M, Masel EK, Neubauer M, Watzke H, et al. Gender differences in caregiver burden and its determinants in family members of terminally ill cancer patients. Psychooncology. 2016;25(7):808–14. 26477788 10.1002/pon.4005

[48] Khalil H, Ristevski E. The challenges of evidence-based palliative care research. Int J Evid Based Healthc. 2018;16(3):136–7. 30148804 10.1097/XEB.0000000000000153

