# Supplementary material for: Effectiveness of interventions for informal caregivers of people with end-stage chronic illness: a systematic review
Source: Syst Rev. 2024 Sep 28;13:245. doi: 10.1186/s13643-024-02641-x (PMC11438131; doi:10.1186/s13643-024-02641-x)
Supplement: Supplementary file 1 — Additional file 1: Supplementary materials. [file 13643_2024_2641_MOESM1_ESM.docx]

**Supplementary Materials**

**Table 1: Database(s): Ovid MEDLINE(R) ALL 
Search Strategy:**

| # | **Searches** |
| --- | --- |
| 1 | exp Chronic Disease/ |
| 2 | Heart Failure/ |
| 3 | renal insufficiency/ or renal insufficiency, chronic/ |
| 4 | Kidney Failure, Chronic/ |
| 5 | Pulmonary Disease, Chronic Obstructive/ |
| 6 | 1 or 2 or 3 or 4 or 5 |
| 7 | (end-stage* or palliative* or dying* or end of life* or terminal* or advanced* or late stage or final stage or stage 4* or stage 5*).mp. |
| 8 | (conservative* or hospice* or home*).mp. |
| 9 | Palliative Care/ |
| 10 | Terminally Ill/ |
| 11 | Hospice Care/ |
| 12 | conservative management.mp. |
| 13 | Conservative Treatment/ |
| 14 | 7 or 8 or 9 or 10 or 11 or 12 or 13 |
| 15 | (family or families or parent* or mother* or father* or friend* or relative* or spouse* or partner* or significant other or husband* or wife or wives or son* or daughter* or offspring or sibling* or brother* or sister*).mp. |
| 16 | (informal* or carer* or caregiv* or care giv*).mp. |
| 17 | Caregivers/ |
| 18 | Family/ |
| 19 | Spouses/ |
| 20 | 15 or 16 or 17 or 18 or 19 |
| 21 | Social Support/ |
| 22 | Family Health/ |
| 23 | exp Psychotherapy/ |
| 24 | exp Counseling/ |
| 25 | ((psycho* or psycholog* or psychosocial) adj intervent*).tw. |
| 26 | Cognitive Behavioral Therapy/ |
| 27 | Relaxation therapy/ |
| 28 | Stress, Psychological/ |
| 29 | Meditation/ |
| 30 | Mindfulness/ |
| 31 | Education/ |
| 32 | Health Education/ |
| 33 | Teaching/ |
| 34 | Self Care/ |
| 35 | psychosocial.mp. |
| 36 | ((support* or inform* or help* or assist* or train* or educat* or teach* or coach* or instruct* or advis* or advice* or counsel* or intervention* or therap* or program*) adj3 (cognitive or psycho* or self help or selfhelp or problem solving or coping or communication or relaxation or self care)).mp. |
| 37 | 21 or 22 or 23 or 24 or 25 or 26 or 27 or 28 or 29 or 30 or 31 or 32 or 33 or 34 or 35 or 36 |
| 38 | 6 and 14 and 20 and 37 |

**Table 2: Measures to assess psychosocial outcomes**

| **Study (Author, Year, Location)** | **Outcome (Measures used) *Measure Author(s)***  **Subscales** |
| --- | --- |
| **Allen, 2008, (1) *USA*** | **Caregiver Stress** (Caregiver Stressors Scale-Revised: CSS-R) *(2)*  **Depression** (Center for Epidemiological Studies- Depression scale: CES-D) (3)  **Psychological wellbeing** (Measure comprised 3 questions measuring perception of life satisfaction; happiness; and goal attainment) (4) |
| **Aloweni, 2022, (5) *Singapore*** | **Caregiver stress** (Perceived Stress Scale: PSS) (6)  **Anxiety** (State-Trait Anxiety Scale: STAI) (7)  State-Trait Anxiety Scale – State Anxiety: STAI-S  State-Trait Anxiety Scale – Trait Anxiety: STAI-T  **Health-Related Quality of Life: HRQoL** (Short-form 36v2: SF-36v2) (8)  Short-form 36 Physical Component Summary: SF-36v2 PCS  Short-form 36 Mental Component Summary: SF-36v2 MCS |
| **Bakitas, 2017, (9) *USA*** | **Caregiver burden** (Montgomery Borgatta Caregiver Burden Scale: MBCB) (10)  MBCB – Total  MBCB – Objective burden  MBCB – Demand burden  MBCB – Stress burden  **Anxiety and Depression** (Hospital Anxiety and Depression Scale: HADS) (11)  Anxiety: Hospital Anxiety and Depression Scale – Anxiety subscale: HADS-A  Depression: Hospital Anxiety and Depression Scale – Depression subscale: HADS-D  **Quality of Life** (Bakas Caregiving Outcomes Scale: BCOS) (12) |
| **Carson, 2016, (13) *USA*** | **Anxiety and Depression** (Hospital Anxiety and Depression Scale: HADS) (11)  Anxiety: Hospital Anxiety and Depression Scale – Anxiety subscale: HADS-A  Depression: Hospital Anxiety and Depression Scale – Depression subscale: HADS-D  **PTSD symptoms** (Impact of Events Scale-Revised: IES-R) (14) |
| **Chan, 2016, (15) *Hong Kong*** | **Caregiver Burden** (Zarit Burden Interview translated: ZBI-t) (16)  **Anxiety and Depression** (Hospital Anxiety and Depression Scale: HADS) (11)  Anxiety: Hospital Anxiety and Depression Scale – Anxiety subscale: HADS-A  Depression: Hospital Anxiety and Depression Scale – Depression subscale: HADS-D |
| **Dionne-Odom, 2020, (17) *USA*** | **Caregiver burden** (Montgomery Borgatta Caregiver Burden Scale: MBCB) (10)  MBCB – Objective burden  MBCB – Demand burden  MBCB – Stress burden  **Anxiety and Depression** (Hospital Anxiety and Depression Scale: HADS) (11)  Anxiety: Hospital Anxiety and Depression Scale – Anxiety subscale: HADS-A  Depression: Hospital Anxiety and Depression Scale – Depression subscale: HADS-D  **Quality of Life** (Bakas Caregiving Outcomes Scale: BCOS) (12) |
| **Douglas, 2005, (18) *USA*** | **Caregiver burden** (Caregiver Reaction Assessment: CRA) (19)  Disrupted Schedule  Finance Concerns  Lack of family support  Physical Health concerns  Self esteem  **Depression** (Center for Epidemiological Studies- Depression scale: CES-D) (3)  **Quality of Life** (Short-form 8: SF-8) (20)  Short-form 8 Physical Component Summary: SF-8 PCS |
| **Gary, 2020, (21) *USA*** | **Caregiver strain** (Measure comprised 2 questions) (21)  **Quality of Life** (Bakas Caregiving Outcomes Scale: BCOS) (12) |
| **Hener, 1996, (22) *Israel*** | **Anxiety** (Mixed scale: comprising items from the Psychological Adjustment to Illness Scale: PAIS - (23); the Brief Symptom Inventory: BSI - (24), and the Millon Behavioral Health Inventory: MBHI - (25)) (22)  **Depression** (Mixed scale: items from the Psychological Adjustment to Illness Scale: PAIS - (23); and the Beck Depression Inventory: BDI – (26)) (22)  Self-efficacy (Self-efficacy scale) (22)  Distress (Social distress scale – 5 items based on the Psychological Adjustment to Illness Scale: PAIS - (23)) (22) |
| **Law, 2021, (27) *Hong Kong*** | **Caregiver strain** (Chinese version of the Modified Caregiver Strain Index: C-M-CSI) (28)  **Psychological Wellbeing** (Measure comprised 1 question) (27) |
| **Liljeroos, 2015, (29) *Sweden*** | **Depression** (Beck Depression Inventory-II: BDI-II) (30)  **Health-Related Quality of Life: HRQoL** (Short-form 36: SF-36) (31)  Short-form 36 Physical Component Summary: SF-36 PCS  Short-form 36 Mental Component Summary: SF-36 MCS |
| **Liljeroos, 2017, (32) *Sweden*** | **Caregiver burden** (Caregiver Burden Scale: CBS) (33) |
| **Sebern, 2012, (34) *USA*** | **Anxiety** (State-Trait Anxiety Scale: STAI) (7)  **Depression** (Patient Health Questionnaire-9: PHQ-9) (35)  **Health-Related Quality of Life: HRQoL** (Rand Short-form 36: Rand SF-36) (36)  SF-36: Physical  SF-36: Emotional  SF-36: Fatigue  SF-36: Pain |

***Figure 4: Overview: Interventions, outcomes, specifics and summary***

*The information in Figure 4 provides an ‘at-a-glance’ overview of the range of interventions reported in each of the included studies. It outlines key aspects considered, including intervention delivery, psychosocial outcomes and effects, as well as reflection on intervention specifics including type, development and participants. A summary of findings is also included.*


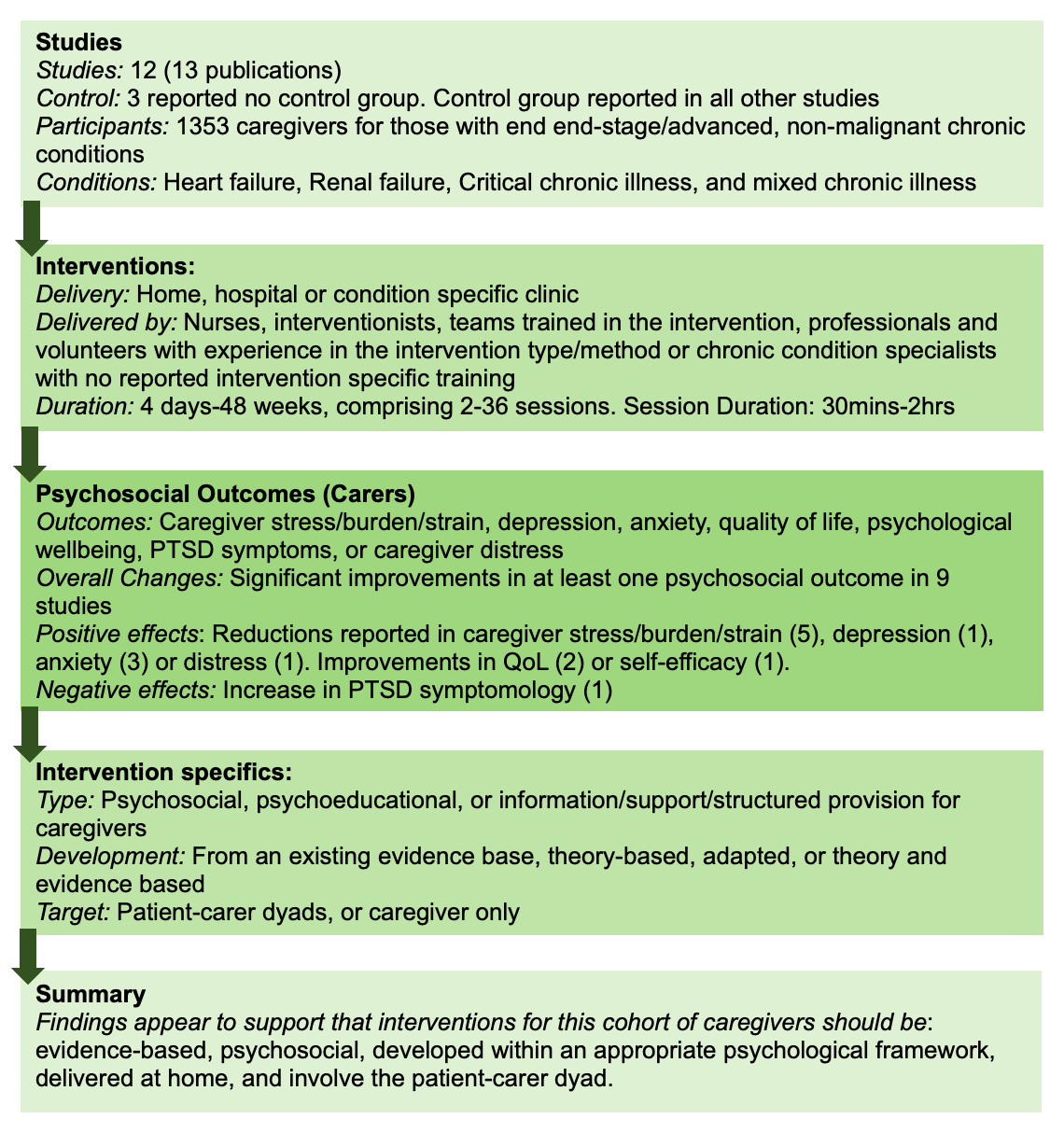


References

1. Allen RS, Hilgeman MM, Ege MA, Shuster Jr JL, Burgio LD. Legacy activities as interventions approaching the end of life. Journal of palliative medicine. 2008;11(7):1029-38.

2. Zarit SH, Stephens MAP, Townsend A, Greene R. Stress reduction for family caregivers: Effects of adult day care use. The Journals of Gerontology Series B: Psychological Sciences and Social Sciences. 1998;53(5):S267-S77.

3. Radloff LS. The CES-D scale: A self-report depression scale for research in the general population. Applied psychological measurement. 1977;1(3):385-401.

4. Tran TV, Wright R, Chatters L. Health, stress, psychological resources, and subjective well-being among older blacks. Psychology and Aging. 1991;6(1):100.

5. Aloweni F, Doshi K, Agus N, Fook-Chong S, Wu SY, Kong LP, et al. Evaluating the feasibility and effectiveness of a mindfulness-based intervention on stress and anxiety of family caregivers managing peritoneal dialysis. Proceedings of Singapore Healthcare. 2022;31:20101058211054913.

6. Cohen S, Kamarck T, Mermelstein R. A global measure of perceived stress. Journal of health and social behavior. 1983:385-96.

7. Spielberger C, Gorsuch R, Lushene R, Vagg P, Jacobs G. Manual for the State-Trait Anxiety Inventory; Palo Alto, CA, Ed. Palo Alto: Spielberger. 1983.

8. Thumboo J, Wu Y, Tai E-S, Gandek B, Lee J, Ma S, et al. Reliability and validity of the English (Singapore) and Chinese (Singapore) versions of the Short-Form 36 version 2 in a multi-ethnic urban Asian population in Singapore. Quality of Life Research. 2013;22:2501-8.

9. Bakitas M, Dionne-Odom JN, Pamboukian SV, Tallaj J, Kvale E, Swetz KM, et al. Engaging patients and families to create a feasible clinical trial integrating palliative and heart failure care: results of the ENABLE CHF-PC pilot clinical trial. BMC palliative care. 2017;16(1):1-13.

10. Montgomery RJ, Gonyea JG, Hooyman NR. Caregiving and the experience of subjective and objective burden. Family relations. 1985:19-26.

11. Zigmond AS, Snaith RP. The hospital anxiety and depression scale. Acta psychiatrica scandinavica. 1983;67(6):361-70.

12. Bakas T, Champion V. Development and psychometric testing of the Bakas Caregiving Outcomes Scale. Nursing research. 1999;48(5):250-9.

13. Carson SS, Cox CE, Wallenstein S, Hanson LC, Danis M, Tulsky JA, et al. Effect of palliative care–led meetings for families of patients with chronic critical illness: a randomized clinical trial. Jama. 2016;316(1):51-62.

14. Weiss DSM, C.R. The impact of event scale-revised. In: J. P. Wilson TMK, editor. Assessing psychological trauma and PTSD. New York: Guilford Press; 1997. p. pp. 399–411.

15. Chan KY, Yip T, Yap DY, Sham MK, Wong YC, Lau VWK, et al. Enhanced psychosocial support for caregiver burden for patients with chronic kidney failure choosing not to be treated by dialysis or transplantation: a pilot randomized controlled trial. American Journal of Kidney Diseases. 2016;67(4):585-92.

16. Chan TS-F, Lam LC-W, Chiu HF-K. Validation of the Chinese version of the Zarit Burden Interview. East Asian Archives of Psychiatry. 2005;15(1):9.

17. Dionne-Odom JN, Ejem DB, Wells R, Azuero A, Stockdill ML, Keebler K, et al. Effects of a telehealth early palliative care intervention for family caregivers of persons with advanced heart failure: the ENABLE CHF-PC randomized clinical trial. JAMA network open. 2020;3(4):e202583-e.

18. Douglas SL, Daly BJ, Kelley CG, O’Toole E, Montenegro H. Impact of a disease management program upon caregivers of chronically critically ill patients. Chest. 2005;128(6):3925-36.

19. Stommel M, Wang S, Given CW, Given B. Focus on psychometrics confirmatory factor analysis (CFA) as a method to assess measurement equivalence. Research in nursing & health. 1992;15(5):399-405.

20. Ware JE, Kosinski M, Dewey JE, Gandek B. How to score and interpret single-item health status measures: a manual for users of the SF-8 health survey. Lincoln, RI; Boston, MA: QualityMetric Incorporated; 2001.

21. Gary R, Dunbar SB, Higgins M, Butts B, Corwin E, Hepburn K, et al. An intervention to improve physical function and caregiver perceptions in family caregivers of persons with heart failure. Journal of Applied Gerontology. 2020;39(2):181-91.

22. Hener T, Matisyohu W, Har-Even D. Supportive versus cognitive–behavioral intervention programs in achieving adjustment to home peritoneal kidney dialysis. Journal of Consulting and Clinical Psychology. 1996;64(4):731.

23. Morrow GR, Chiarello RJ, Derogatis LR. A new scale for assessing patients' psychosocial adjustment to medical illness. Psychological Medicine. 1978;8(4):605-10.

24. Derogatis LR, Melisaratos N. The brief symptom inventory: an introductory report. Psychological medicine. 1983;13(3):595-605.

25. Millon T, Green CJ, Meagher RB. The MBHI: A new inventory for the psychodiagnostician in medical settings. Professional Psychology. 1979;10(4):529.

26. A.T. B. Depression Inventory. Philadephia: Center for Cognitive Therapy; 1978.

27. Law M-C, Lau BH-P, Kwok AY, Lee JS, Lui RN, Liu K, et al. Empowering families facing end-stage nonmalignant chronic diseases with a holistic, transdisciplinary, community-based intervention: 3 months outcome of the Life Rainbow Program. Palliative & supportive care. 2021;19(5):530-9.

28. Chan WCH, Chan CL, Suen M. Validation of the Chinese version of the Modified Caregivers Strain Index among Hong Kong caregivers: An initiative of medical social workers. Health & Social Work. 2013;38(4):214-21.

29. Liljeroos M, Ågren S, Jaarsma T, Årestedt K, Strömberg A. Long term follow-up after a randomized integrated educational and psychosocial intervention in patient-partner dyads affected by heart failure. PLoS One. 2015;10(9):e0138058.

30. Beck AT, Steer RA, Brown G. Beck depression inventory–II. Psychological assessment. 1996.

31. Ware JE, Kosinski, M., & Keller, S. D. SF-36 physical and mental health summary scales: a user's manual. Boston: The Health Institute, New England Medical Center; 1994.

32. Liljeroos M, Ågren S, Jaarsma T, Årestedt K, Strömberg A. Long-term effects of a dyadic psycho-educational intervention on caregiver burden and morbidity in partners of patients with heart failure: a randomized controlled trial. Quality of Life Research. 2017;26:367-79.

33. Elmståhl S, Malmberg B, Annerstedt L. Caregiver's burden of patients 3 years after stroke assessed by a novel caregiver burden scale. Archives of physical medicine and rehabilitation. 1996;77(2):177-82.

34. Sebern MD, Woda A. Shared care dyadic intervention: outcome patterns for heart failure care partners. Western journal of nursing research. 2012;34(3):289-316.

35. Kroenke K, Spitzer RL, Williams JB. The PHQ‐9: validity of a brief depression severity measure. Journal of general internal medicine. 2001;16(9):606-13.

36. Ware Jr JE, Sherbourne CD. The MOS 36-item short-form health survey (SF-36): I. Conceptual framework and item selection. Medical care. 1992:473-83.
